# Reactant Discovery with an *Ab Initio* Nanoreactor: Exploration of Astrophysical N-Heterocycle Precursors and Formation Pathways

**DOI:** 10.1021/acsearthspacechem.4c00120

**Published:** 2024-08-09

**Authors:** Sommer L. Johansen, Heejune Park, Lee-Ping Wang, Kyle N. Crabtree

**Affiliations:** Department of Chemistry, University of California, Davis, Davis, California 95616, United States

**Keywords:** astrochemistry, molecular dynamics, potential energy surfaces, method development, reaction kinetics

## Abstract

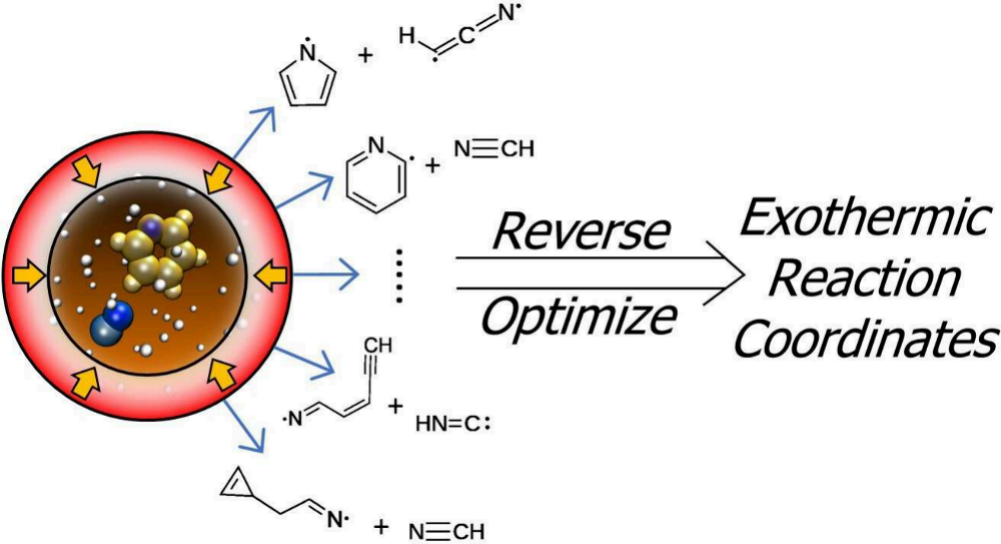

The incorporation of nitrogen atoms into cyclic compounds is essential for terrestrial life; nitrogen-containing (N-)heterocycles make up DNA and RNA nucleobases, several amino acids, B vitamins, porphyrins, and other components of biomolecules. The discovery of these molecules on meteorites with non-terrestrial isotopic abundances supports the hypothesis of exogenous delivery of prebiotic material to early Earth; however, there has been no detection of these species in interstellar environments, indicating that there is a need for greater knowledge of their astrochemical formation and destruction pathways. Here, we present results of simulations of gas-phase pyrrole and pyridine formation from an *ab initio* nanoreactor, a first-principles molecular dynamics simulation method that accelerates reaction discovery by applying non-equilibrium forces that are agnostic to individual reaction coordinates. Using the nanoreactor in a retrosynthetic mode, starting with the N-heterocycle of interest and a radical leaving group, then considering the discovered reaction pathways in reverse, a rich landscape of N-heterocycle-forming reactivity can be found. Several of these reaction pathways, when mapped to their corresponding minimum energy paths, correspond to novel barrierless formation pathways for pyridine and pyrrole, starting from both detected and hypothesized astrochemical precursors. This study demonstrates how first-principles reaction discovery can build mechanistic knowledge in astrochemical environments as well as in early Earth models such as Titan’s atmosphere where N-heterocycles have been tentatively detected.

## 1 Introduction

In 1828, Friedrich Wöhler published the synthesis of urea, a product of the activities of living organisms, from cyanic acid and ammonia.^1,2^ This discovery posed a direct challenge to vitalism, which is the belief that chemical compounds related to “life” can only be produced by living organisms. The surprising disclosure opened a new era for exploring the origin of life.^2^ Since the journey began, the origin of nucleobases, heterocyclic compounds containing nitrogen atoms in their rings (N-heterocycles), has been receiving great attention as they are the building blocks of genetic materials of all living organisms.^3−5^

Astronomers and astrobiologists have been studying the potential interstellar formation of N-heterocycles.^5^ A wide variety of species that contain this motif have been identified on meteorites with non-terrestrial isotopic abundances,^6^ and there is evidence that the 6.2 μm unidentified infrared (UIR) band can only arise from the presence of a nitrogen atom in a polycyclic aromatic hydrocarbon (PAH) structure.^7^ However, despite the 124 nitrogen-containing species identified in space (per the Cologne Database of Molecular Spectroscopy^8^ at the time of this writing) all astronomical searches for N-heterocycles have been unsuccessful.^9−11^

Recently, cyano (CN)-substituted cyclic carbon rings have been discovered in the Taurus Molecular Cloud-1 (TMC-1).^12^ This cold molecular cloud has a temperature near 10 K and number density of order 10^3^ cm^–3^. Despite these conditions, reactions of aromatics and CN have been shown to proceed rapidly due to a barrierless reaction coordinate.^13^ The derived abundances of recently detected aromatic and cyclic molecules greatly exceed the values predicted by chemical models, despite their accurate predictions of acyclic carbon chain abundances. This survey included a search for N-heterocycles pyrrole (c-C_4_H_5_N) and pyridine (c-C_5_H_5_N), but they were not found; the column density upper limits were ∼10^12^ cm^–2^, comparable to the detected abundances of indene^14^ and cyanonaphthalene.^15^ If homocyclic carbon rings and N-heterocycles are formed in such a low-temperature environment through bottom-up chemistry, the pathways must proceed through different classes of reactions or require precursors that exist in abundance for the homocyclic but not heterocyclic pathways. Overall, this indicates that there is a wealth of low-temperature astrochemistry that we currently do not understand.

N-heterocycles are also of significant interest in the study of Titan’s atmosphere. The Cassini/Huygens mission made several tentative detections of single-ringed species, along with a wealth of other N-containing complex organic molecules that could be potential N-heterocycle precursors.^16−18^ Titan is considered an analog for early Earth,^19−21^ so understanding the role N-heterocycles play in this environment could provide insight into potential terrestrial formation of such prebiotic species.

**Figure 1:**
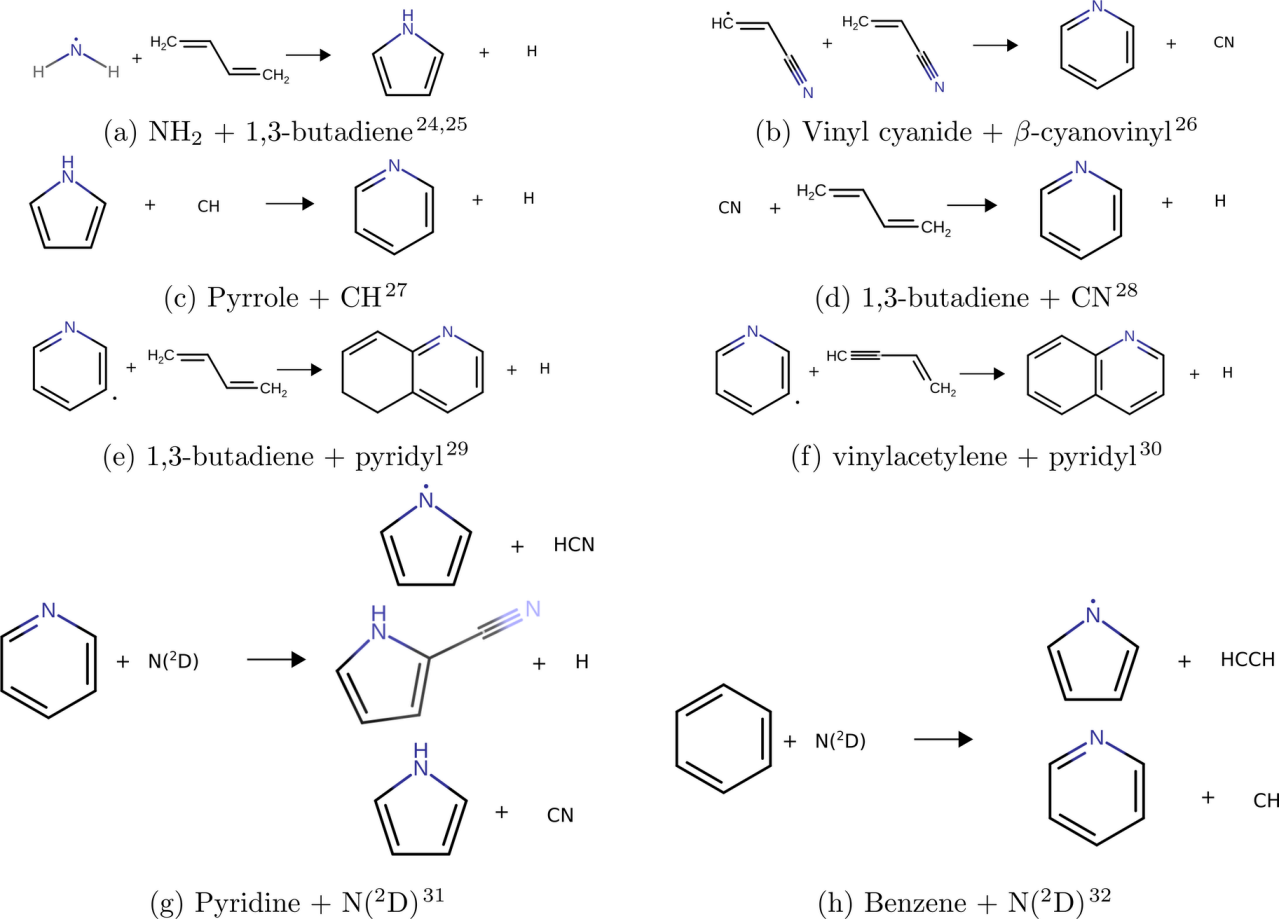
Gas-phase, neutral–neutral N-heterocycle formation pathways.

**Figure 2:**
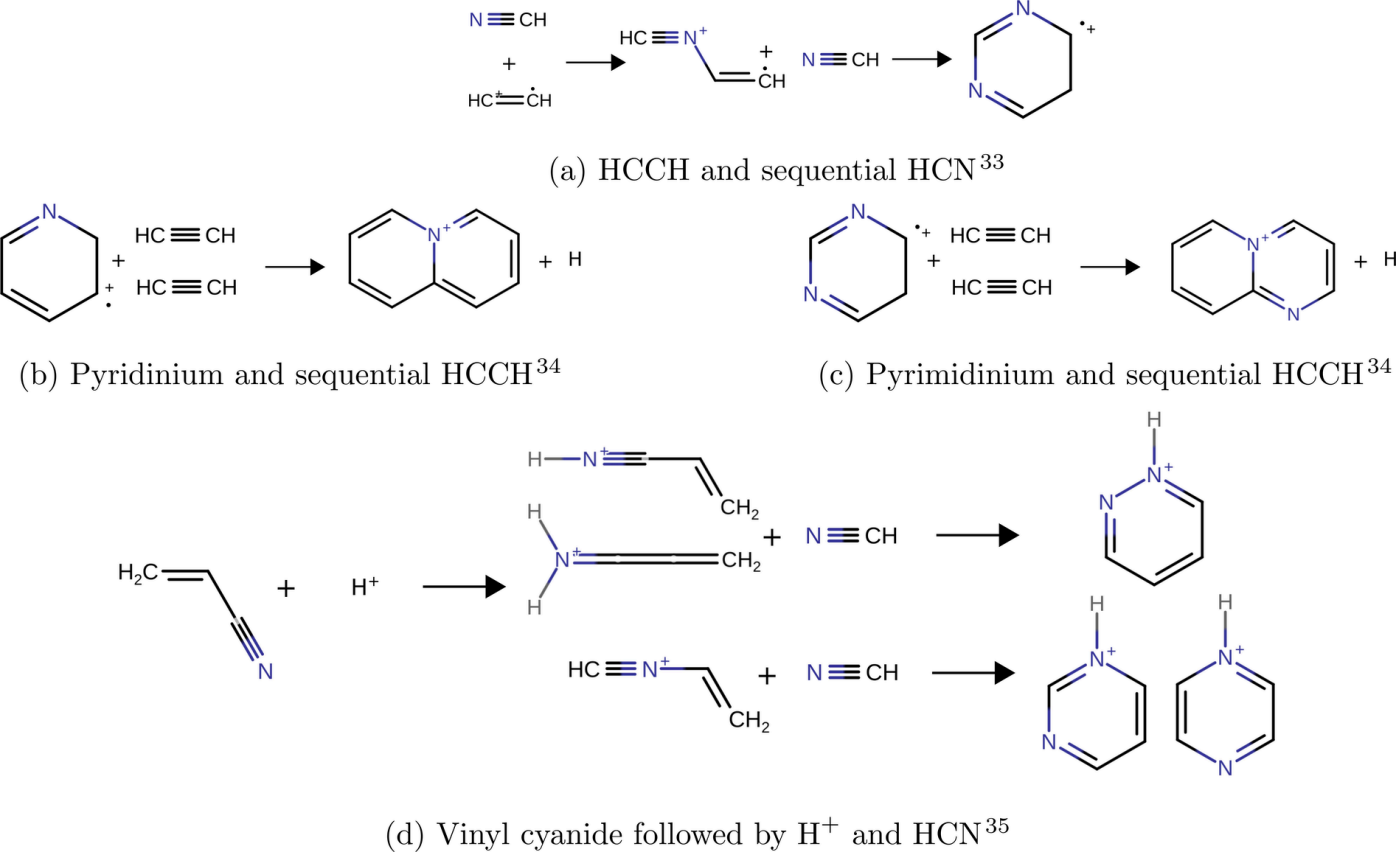
Gas-phase, ion–neutral N-heterocycle formation pathways.

Ultimately, an understanding of how N-heterocycles participate in meteoritic chemistry, nitrogen-containing polycyclic aromatic hydrocarbon (PANH) formation, and Titan’s atmosphere requires inclusion of appropriate formation and destruction pathways into chemical kinetics models. These models seek to replicate the chemistry of a particular environment, and when done comprehensively, can offer accurate predictions of molecular abundances. However, few low-temperature pathways that include N-heterocycles are available in the current literature. To illustrate this, Figure 1 includes, to the best of our knowledge, all astrochemically viable neutral–neutral N-heterocycle forming reactions proposed in the literature, while ion–neutral formation pathways are shown in Figure 2. Neither KIDA nor UMIST, databases of chemical reactions relevant for astrochemistry, include any reactions of N-heterocycles.^22,23^ More reactions must be identified in order to accurately incorporate N-heterocycles within chemical kinetics models.

Computational reaction discovery methods, which automate the generation and testing of mechanistic hypotheses, can play an important role in determining new reaction pathways for N-heterocycle formation and other important astrochemical questions. These methods employ a variety of strategies for hypothesis generation, which include the systematic driving of all possible combinations of reaction coordinates derived from graph-theoretical representations,^36−51^ or carrying out *ab initio* molecular dynamics (AIMD) simulations [or other types of reactive molecular dynamics (MD)] which contain “reaction events” in the simulation trajectory.^52−62^ The generated mechanistic hypotheses are tested by computing the potential energy surface (PES) critical points followed by modeling the reaction rates, which filters the discovered pathways down to the kinetically viable ones. Such approaches have found success in gas-phase chemistry including astrochemically relevant reactions,^63,64^ organometallic catalysis,^65,66^ surface chemistry,^67,68^ photochemistry,^69^ and prebiotic chemistry.^53,61,70−73^

This study uses the *ab initio* nanoreactor,^53,54^ a reaction discovery method that combines an AIMD simulation with a time-dependent external potential that induces high-velocity molecular collisions and provides the kinetic energy needed to cross over activation barriers on the relatively short AIMD-accessible time scales. An advantage of the nanoreactor is that it does not require user-specified reaction coordinates, and thus enables discovery of reactions within a broader space than one defined by rules; the downside is an increased computational cost and the inability to exhaustively sample all pathways. The nanoreactor is an ideal choice for this work because astrochemical reaction pathways can transcend traditional chemical intuition, as demonstrated by the prevalence of radicals, gas-phase ions, highly unsaturated molecules, carbenes and other exotic species not common to terrestrial chemistry. A key aspect of this work is that the nanoreactor simulations are carried out to discover the pathways of retrosynthesis,^74^ that is, to find endothermic and barrierless fragmentation pathways, which correspond to kinetically feasible synthesis pathways of N-heterocycles under ultracold astrochemical conditions.

Here we detail this search for pyridine and pyrrole formation mechanisms. First, the nanoreactor simulations and the wide range of N-heterocycle breakdown pathways are described. Then, we discuss novel reactions found by expanding upon pathways identified in the nanoreactor simulations: first, a pyrrole forming pathway with β-cyanovinyl (HCCHCN) and ethenimine (H_2_CCNH) precursors, followed by two reaction schemes whereby a *n*-membered ring is formed through an insertion reaction into a (*n* – 1)-membered ring; specifically, the formation of pyrrole from azete (c-C_3_H_4_N) and cyanomethyl (H_2_CCN), and the formation of pyridine from 1-pyrrolyl (c-C_4_H_4_N) and cyanomethylene (HCCN).

## 2 Methods

The computational approach employed here consists of three stages: “discovery”, “selection”, and “refinement”. The discovery stage involves the use of the *ab initio* nanoreactor method,^53^ which employs AIMD simulations at high temperature with an additional time-dependent external potential that induces high-velocity molecular collisions, thereby accelerating reactivity. The trajectories of reactions occurring during the simulation are extracted and a subset of reactions are then selected for further analysis on the basis of their plausibility as astrophysical routes to N-heterocycle formation. The refinement stage includes searching for minimum energy paths (MEPs) for selected reactions discovered in the nanoreactor. Following the refinement stage, the results were further expanded by carrying out potential energy surface exploration starting from model structures inspired by the nanoreactor results.

To begin the discovery stage, initial configurations of molecules were made using the program Packmol,^75^ which populates molecules and atoms of interest within spheres of certain radii without overlapping atoms. These spheres contained either pyridine and CN or pyrrole and CN along with a number of inert He atoms (between 10 and 50) in various initial configurations (see Table S1 of the Supporting Information). In principle any inert atom could be used as an energy transfer agent in the simulations; we choose helium to minimize the number of electrons that need to be treated in the ab initio calculations. In total, 405 AIMD simulations were carried out with various sets of the parameters (*R*_1_, *R*_2_, *t*_1_, *t*_2_, *k*_1_, and *k*_2_). Table S2 of the Supporting Information presents ranges of these parameters for each labeled simulation. All simulations began with either pyrrole or pyridine and CN, in order to search for mechanisms that result in the desired N-heterocycle with CN as a byproduct. Each starting configuration was energy-minimized^76,77^ prior to starting nanoreactor MD simulations. Both the minimizations and nanoreactor simulations were carried out with the B3LYP hybrid density functional, an unrestricted Kohn–Sham wave function and the 6-31G(d) Gaussian basis set, as implemented in the TeraChem^78^ quantum chemistry software.

The characteristic feature of the nanoreactor simulation is the time-dependent boundary potential which induces reactivity
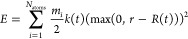
1where
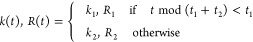
2This potential is zero within a spherical region of radius *R*(*t*) and is harmonic outside this region with a force constant *m*_*i*_*k*(*t*); the mass term ensures uniform acceleration of molecules subject to forces from the boundary potential. *R*(*t*) and *k*(*t*) oscillate between set values *R*_1_, *k*_1_ and *R*_2_ < *R*_1_, *k*_2_ in a rectangular wave pattern with upper and lower periods of *t*_1_ and *t*_2_. The *t*_2_ interval is analogous to the downward stroke of a piston that pushes the molecules into the smaller *R*_2_ sphere where reactivity is initiated by high-energy collisions between the He atoms and the starting species. Afterward, during the *t*_1_ interval the sphere radius reverts to *R*_1_ and the molecules are free to diffuse into the larger volume. The cycle repeats during the course of the simulation. See Figure S1 of the Supporting Information for a visual example of this process.

The time step was set to 0.5 fs and a Langevin thermostat was used with an equilibrium temperature between 1000 and 3500 K and a damping time of 300.0 fs. Table S2 of the Supporting Information details the range of parameters explored for each starting configuration. Each simulation was allowed to run for 24 h of wall time, yielding several picoseconds of simulation time.

The second stage of this process is the “selection” of reactions to include in the following “refinement” stage. Reactions were detected in the simulation trajectories using an automated trajectory analysis method^54^ that constructs molecular graphs from interatomic distances and detects reaction events corresponding to lasting changes in connectivity. The results of the trajectory analysis were confirmed by visualization with the VMD program.^79^

Reactions identified in the nanoreactor simulations are then filtered based on their plausibility under astrophysical conditions in an interstellar cloud: low temperature (≤20 K) and density (≤10^7^ cm^–3^).^80^ The first selection criterion is that the overall reaction must be bimolecular, as three-body collisions are exceedingly improbable in these environments. Similarly, the low density and temperature preclude spontaneous unimolecular rearrangements, so such processes are rejected except those that occur along a bimolecular pathway via submerged barriers. The second criterion is that the overall reaction coordinate is exothermic: the precursors must be higher in energy than the products. Finally, we prioritized reactions involving precursors that were non-intuitive and/or had not been proposed previously in other studies of N-heterocycle formation.

During the refinement stage, reaction events of interest are further investigated at a more accurate level of theory to determine their kinetic feasibility. The refinement procedure starts with energy minimization of the intermediates extracted from the nanoreactor AIMD trajectory, then transition state (TS) structures on the minimum energy paths connecting the intermediates are located using the nudged elastic band (NEB) method. NEB calculations are initiated by linear interpolation of frames from the nanoreactor simulations connecting two optimized intermediates. The highest-energy structures are then used as inputs to TS optimization calculations to find the precise first order saddle points. Harmonic frequency calculations are performed on all structures to compute the zero point vibrational energy (ZPVE) and confirm the presence of one imaginary mode for the TS structures. The connections between the optimized TS and its adjacent energy basins are verified using the intrinsic reaction coordinate method. The energy minimization, NEB, and harmonic frequency calculations were performed using the range-separated ωB97X-D3^81^ DFT functional, an unrestricted KS wave function, and the cc-pVTZ basis set as implemented in the Q-Chem quantum chemistry software.^82^ To provide more accurate PES landscapes along the reaction coordinates, single-point energy calculations for all the optimized geometries are carried out using the CCSD(T)/cc-pVTZ level of theory. The ZPEs of CCSD(T) are obtained by adding ZPVEs calculated with ωB97X-D3 DFT functional to the single-point CCSD(T) electronic energies. Special attention was given to prereaction complexes and searches for barriers along the entrance channel. The prereaction complexes were obtained by optimizing the two reactant molecules using the broken-symmetry approach. Before the optimization, the two reactant molecules were oriented in a way that they have the same relative orientation as the first intermediate. They were separated with a distance larger than 5.00 Å and the optimization was carried with a maximum step size of 0.015 Å. The initial barriers were then searched through sequential constrained optimizations while scanning along the selected interatomic distance.

## 3 Results and Discussion

### 3.1 Nanoreactor Simulations

Out of the 405 nanoreactor simulations that were carried out, the simulations started from a single N-heterocycle and one CN^•^ produced the greatest number of bimolecular pathways. In what follows, we will focus exclusively on the results from these simulations and discuss their relevance for astrophysical environments and planetary atmospheres. Generally, the *R*_1_ and *k*_2_ parameters influenced the simulation outcome most significantly, likely because increasing the expanded sphere size (*R*_1_) and force constant of the contracted sphere (*k*_2_) both contribute directly to increasing the kinetic energy of molecular collisions.

#### 3.1.1 Pyridine

**Figure 3:**
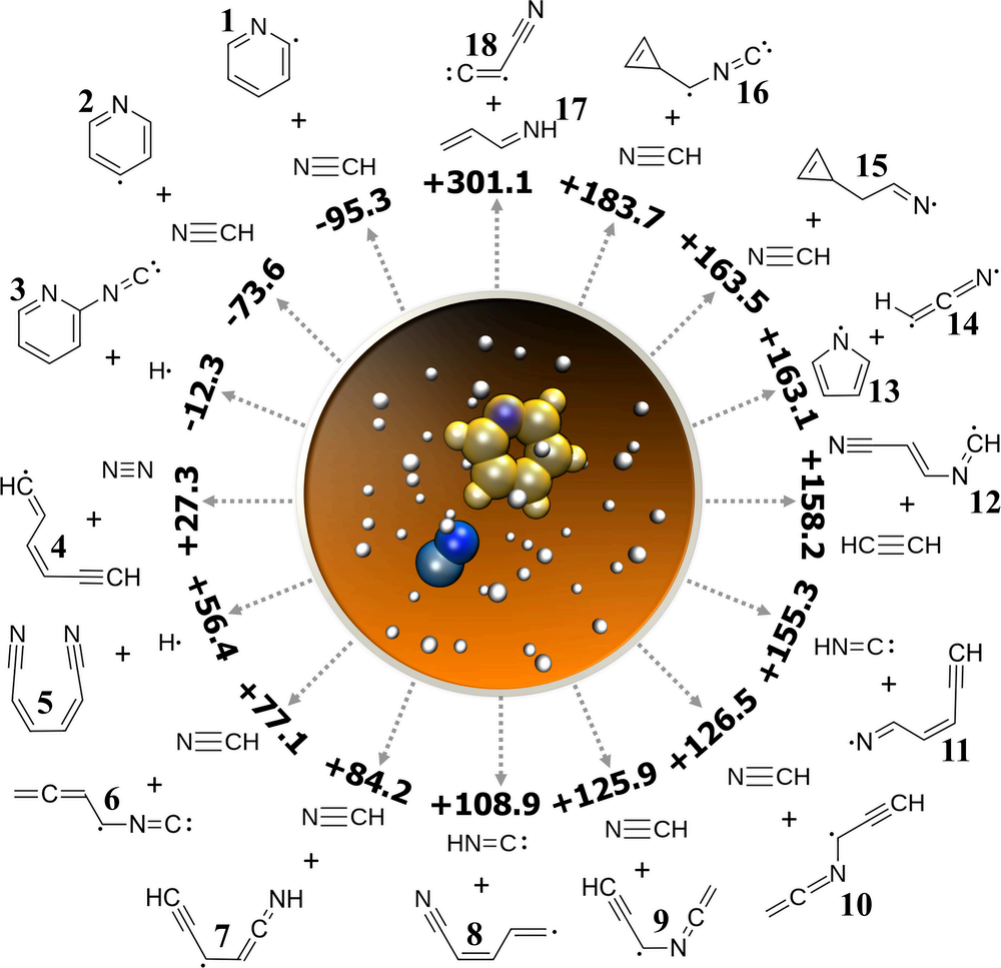
Products from pyridine + CN^•^ reaction. Electronic energy differences are in kJ/mol.

A total of 76 pyridine + CN^•^ nanoreactor simulations were carried out using four different initial configurations of the pyridine, CN, and helium atoms. Bimolecular reactions were observed in 19 out of 76 simulations, with several trajectories leading to the same products; the simulation parameters are listed in Table S3 of the Supporting Information. Figure 3 presents the 16 unique products along with their energies relative to the energy of the reactants, pyridine and CN.

The reaction pyridine + CN^•^ → pyrrolyl + HCCN was observed twice in separate simulations. In both cases, CN^•^ first forms a bond with the pyridine C at the *ortho* C position, then extracts the *ortho* carbon from the ring; when viewed in reverse, this is a possible mechanism for N-heterocycle ring expansion. This reaction was chosen as an example for further refinement, the results of which are discussed in section 3.2.3.

The other 15 reactions in this group were not further refined in this study, but due to their potential astronomical interest, we describe their general characteristics here. Three of the simulations went energetically downhill and left the pyridine ring intact; two of them are characterized by H transfer from pyridine to CN^•^ resulting in formation of pyridyl radicals **1** and **2**, and a third simulation resulted in *ortho*-isocyanopyridyl **3** + H. The other 12 simulations went energetically uphill and are potential candidates for barrierless, pyridine-forming reactions in reverse. Eight trajectories produced a C_5_H_4_N radical that was linear (**6**–**11**) or contained a cyclopropene moiety (**15** and **16**) plus a HCN or HNC co-product. Closed-shell molecules have been detected in the ISM with strong structural resemblance to these radicals: most notably, *trans*-cyanovinylacetylene (HCCCHCHCN)^83^ differs from radicals **7**, **8**, and **11** by the addition of only one hydrogen atom. Additionally, rotational spectra have been measured for three cyanobutadiene isomers, including the parent species of radical **8**, which could enable further astronomical detections.^84^ Other structurally similar molecules found in the ISM include propargyl cyanide (HCCCH_2_CN),^85^ vinylcyanoacetylene (H_2_CCHCCCN)^83^ and the 3-membered rings cyclopropenylidene,^86^ cyclopropenone,^87^ and ethynyl cyclopropenylidene. Cyclopropenylidene has also been detected in Titan’s atmosphere.^17^

The four simulations that went energetically uphill without producing HCN/HNC are described as follows: In one simulation, we observed the formation of product **5** (1,4-dicyanobutadiene), which differs from the recently detected 1-cyanobutadiene^88^ by the addition of one cyano functional group. Another simulation formed the linear radical **12**, a H adduct of 3-isocyanoacrylonitrile, and acetylene C_2_H_2_;^89−92^ the former is a CN adduct of the detected molecule vinyl cyanide.^93^ The species **5** and **12** are also similar to other N-substituted unsaturated organic molecules that have been detected in the ISM including isocyanogen (CNCN),^94^ E- and Z-cyanomethanimine (HNCHCN),^95,96^ aminoacetonitrile (H_2_NCH_2_CN),^97^ and others.^98−100^ One simulation resulted in the pair of species 1-azabutadiene (H_2_CCHCHNH, **17**) and cyanoethynyl radical (C_3_N, **18**): the former is an isomer of 2-azabutadiene which was recently characterized spectroscopically and highlighted as a molecule of astrochemical significance^101^ and the latter is a known molecule in the ISM.^102,103^ Lastly, one trajectory produced N_2_ and the unsaturated linear radical **4**, which is a structural isomer of phenyl radical and related to butadienylacetylene (H_2_CHCHCHCCH) by the removal of H. While butadienylacetylene has not been detected in the ISM to date, it has a similar length and degree of saturation as the known astromolecules vinyl acetylene (H_2_CCHCCH),^104^ allenyl acetylene (H_2_CCCHCCH)^105^ and allenyl diacetylene (H_2_CCCHC_4_H).^106^

Overall, the outcomes of the pyridine + CN^•^ nanoreactor simulations could be characterized in the following ways: (1) The majority of bimolecular products were energetically uphill, indicating they could be candidates for barrierless formation pathways of pyridine + CN^•^ in reverse. (2) A large number of reactions resulted in ring-opening of pyridine to unsaturated linear molecules, which are chemically and structurally very close to molecules detected in the ISM. (3) A smaller number of reactions were energetically downhill which suggest potential reactions of pyridine in the ISM. Interestingly, CN substitution reactions, which have been recently calculated to be barrierless and exothermic,^107^ were not observed in the nanoreactor simulations. Hydrogen abstraction and NC substitution were observed instead even though these reactions are calculated to have barriers, highlighting the contrast between “reverse” nanoreactor simulations and traditional PES exploration. These results point to multiple possible pathways for pyridine formation from unsaturated linear precursors, and demonstrate the effectiveness of the nanoreactor for generating astrochemical reaction hypotheses.

#### 3.1.2 Pyrrole

**Figure 4:**
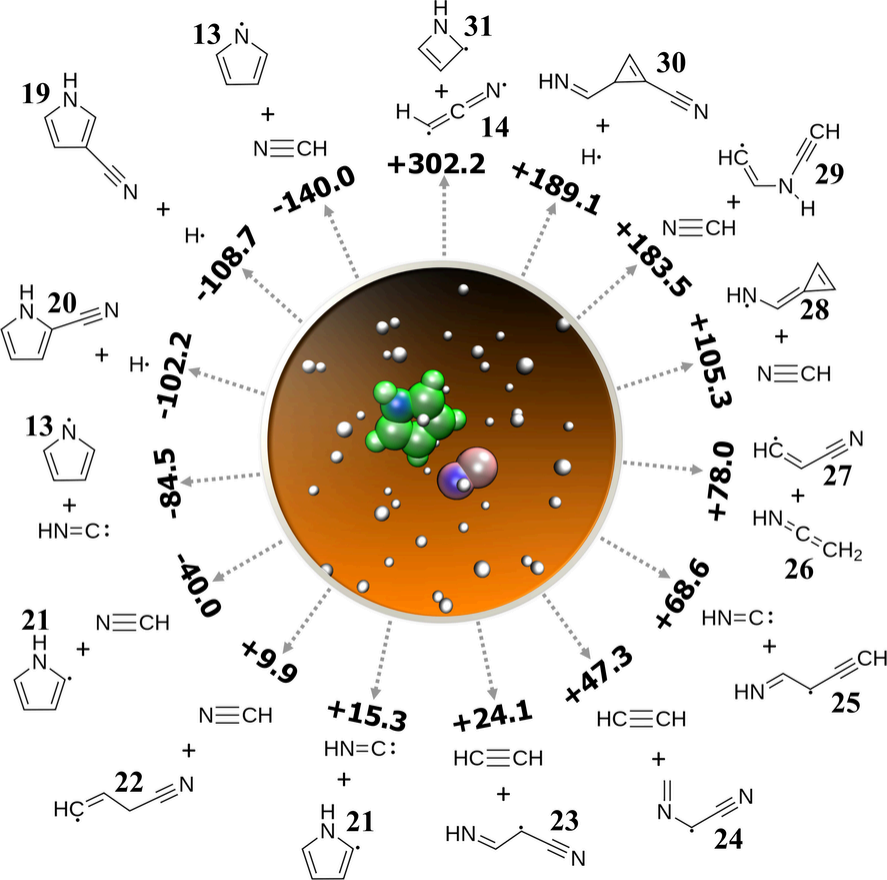
Products from pyrrole + CN^•^ reaction. Electronic energy differences are in kJ/mol.

A total of 60 pyrrole + CN^•^ simulations were performed with 6 different initial configurations. Initial minimization resulted in CN bound to the ring: 5 configurations led to binding at the 2 position, adjacent to N (one of which was in an isocyano configuration), and the other was at the 3 position. Among these, 17 of the 60 simulations led to the formation of two products as shown in Figure 4 and in Table S4 of the Supporting Information. Three simulations led to 1-pyrrolyl (**13**) + HCN/HNC, and the rest led to unique species. Of the reactions observed, 10 were endothermic, meaning that the reverse reactions are energetically favorable for the formation of pyrrole.

Perhaps the two most interesting reverse reactions discovered were ethenimine **26** + β-cyanovinyl **27** and **14** + 1-*H*-2-azetyl **31**. **26** + **27** is quite similar to the reaction **27** + vinyl cyanide (C_3_H_3_N) → pyridine + CN that has been observed under single-collision conditions in a crossed molecular beam experiment and which was calculated to be barrierless using CCSD(T)/cc-pVTZ//B3LYP/cc-pVTZ.^29^ In both cases, the reaction proceeds by addition of the radical **27** at the CH_2_ carbon, followed by a rearrangement and cyclization prior to the elimination of CN. The reaction **14** + **31** is a ring expansion reaction similar to the aforementioned pyridine-forming reaction of radicals **13** + **14**. Both of these pathways were selected for further refinement, discussed in detail below.

Of the remaining pathways, all that proceeded downhill left the 5-membered ring structure intact; they were either hydrogen abstraction reactions leading to HCN/HNC or CN-substitution reactions. Reactions of pyrrole with CN do not appear to have been previously studied, but the analogous formation of benzonitrile from benzene + CN is known to be barrierless, while the H-abstraction channels possess a large barrier.^13^ In contrast, only one of the endothermic reactions preserves the ring: the hydrogen abstraction leading to 2-pyrrolyl **21** + HNC. In addition to radical **31**, two other ring-containing species were found, both forms of functionalized cyclopropene: 3-iminomethyl-1-cyanocyclopropene **30** and cycloprop-2-en-1-ylidenemethanaminyl radical **28**. Neither species has been previously studied; however, the latter is structurally similar to (cyanomethylene)cyclopropane, recently characterized by rotational spectroscopy and an excellent candidate for astronomical searches due to the recent detections of other cyano-substituted species.^108^

The remaining pathways involved breaking the pyrrole structure into acyclic fragments: one of which is closed-shell (C_2_H_2_, HCN, or HNC) and the other is open-shell (**23**, **24**, **25**, and **29**). These open-shell molecules are all related to known astronomical molecules by addition of a small functional group and removal of a hydrogen atom. Two of these are related to ethanimine^109^ by a substitution and H atom removal on the terminal carbon: radical **23** is functionalized with CN, and radical **25** is functionalized with C_2_H, both common functionalization patterns among molecules detected in space. Radical **24** is a CN-functionalized radical derivative of *N*-methylmethanimine, closely related to methanimine,^110^ and radical **29** is a C_2_H-functionalized radical derivative of vinylamine.^111^ In reverse, each of these pathways presents a potential formation route to pyrrole via cyclization and elimination of CN, and the parent species of radicals **23**, **24**, **25**, and **29** are intriguing targets for astronomical detection. Among these, rotational spectra for the parent species of radical **24**(112) and a branched isomer of the parent of radical **23**(113) have been measured to date.

### 3.2 Pathway Refinement and Additional Potential Energy Surface Exploration

Following the initial nanoreactor simulations, three pathways matching the filtering criteria discussed in section 2 were further refined and expanded to explore the mechanisms of the “reverse” reactions and to locate any barrier(s) along the reaction coordinate. For the pyridine + CN simulation, this was the pathway leading to radicals **13** + **14**, and for pyrrole + CN, those leading to products **14** + **31** and **26** + **27**. In the case of products **14** + **31**, initial exploration of the PES led to identification of a lower energy bimolecular configuration than the final product in the nanoreactor trajectory. H atom transfer from radical **31** to radical **14** leads to azete (c-C_3_H_3_N) + cyanomethyl (CH_2_CN) approximately 200 kJ/mol lower in energy; this was chosen as the starting point for the detailed PES as discussed further in section 3.2.1. The other two pathways were explored starting directly from products identified in the nanoreactor simulations: the reaction **26** + **27** → pyrrole + CN is discussed in section 3.2.2 and the reaction **13** + **14** → pyridine + CN in section 3.2.3.

#### 3.2.1 Azete + Cyanomethyl Radical

**Figure 5:**
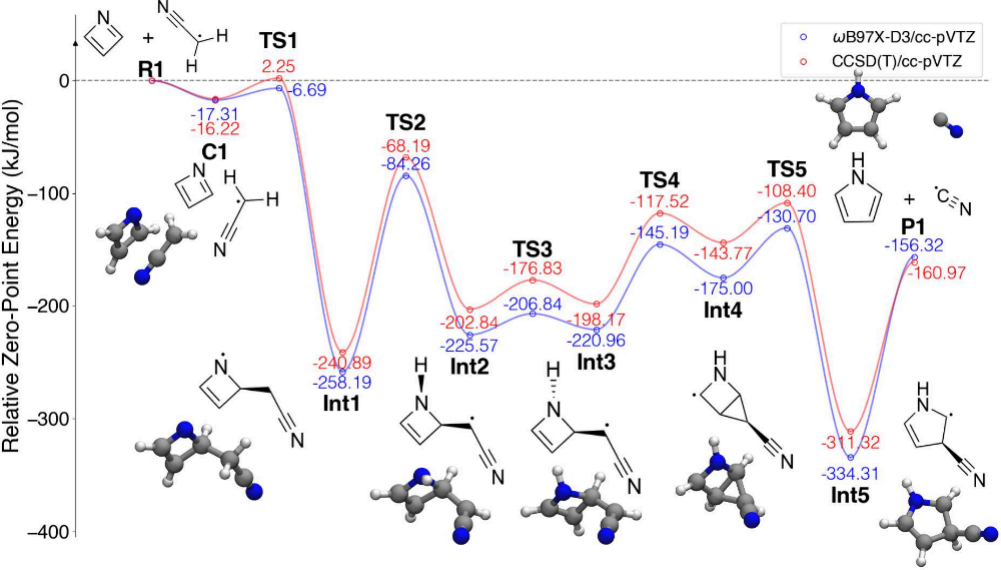
PES detailing the reaction of azete (c-C_3_H_3_N) with the cyanomethyl radical (H_2_CCN^•^). Curves connecting points are fitted lines, not an actual shape of the PES.

The PES for the reaction azete + cyanomethyl → pyrrole + CN is shown in Figure 5. This calculation was performed on a doublet surface to reflect the spin multiplicity of the products: azete was optimized as a singlet and cyanomethyl as a doublet. Azete is an example of an antiaromatic 4π-electron system like cyclobutadiene, but the nitrogen substitution in azete breaks the *D*_4*h*_ symmetry and lifts the degeneracy of the two non-bonding π orbitals. Early multireference configuration interaction calculations with a 4-31G basis set found a that the ground-state electronic structure of azete is well-described by singlet closed-shell wave function with little diradical character owing to the electronegativity difference between the C and N atoms located at opposite vertices of the ring.^114^ This is reflected by the much shorter N=C bond compared with the C=C bond across the ring. A more recent CCSD(T) structural study with basis sets cc-pVTZ, cc-pVQZ, cc-pwCVTZ, and cc-pwCVQZ described azete as a diradical,^115^ but details of the electronic structure were not explicitly addressed and the structure is consistent with that of ref (114). The ωB97X-D3/cc-pVTZ structure here agrees well with that in ref (115), with bond lengths differing by no more than 0.01 Å.

In the reaction, azete and H_2_CCN^•^ first form a pre-reaction complex (**C1**). Both the DFT and CCSD(T) calculations show that complex **C1** is below the zero-point energy (ZPE) of separate reactants **R1**. The entrance barrier (**TS1**) calculated with DFT is submerged under the ZPE of reactants **R1**, whereas the CCSD(T) result presents a barrier that is 2.25 kJ/mol above. Through the entrance barrier, complex **C1** forms a bond between the terminal C of H_2_CCN^•^ and the α C of azete (**Int1**). A H atom transfer then occurs between the newly bonded C of H_2_CCN and the azete N to produce intermediate **Int2**. In the next step to intermediate **Int3**, the N–H bond flips downward, away from the HCCN group, followed by formation of a second bond between the bonded C in the HCCN chain and the adjacent azete C to make the bicyclic structure **Int4**. Ring expansion leads to the 5-membered ring **Int5**, the cyano-pyrrole radical. The final products pyrrole and CN^•^ are then reached by dissociation.

H_2_CCN has been detected in the ISM in TMC-1 and Sgr B2,^116^ and is presumed to be present in Titan’s atmosphere based on data from the Cassini/Huygens INMS and current chemical models.^117−120^ Azete, on the other hand, has neither been isolated nor detected spectroscopically, potentially due to its high reactivity. Indirect evidence exists for its formation via photolysis of azapyrones in Ar matrices;^121^ whether it could be formed via astrochemically relevant gas-phase pathways remains an open question.

#### 3.2.2 Ethenimine + β-Cyanovinyl Radical

**Figure 6:**
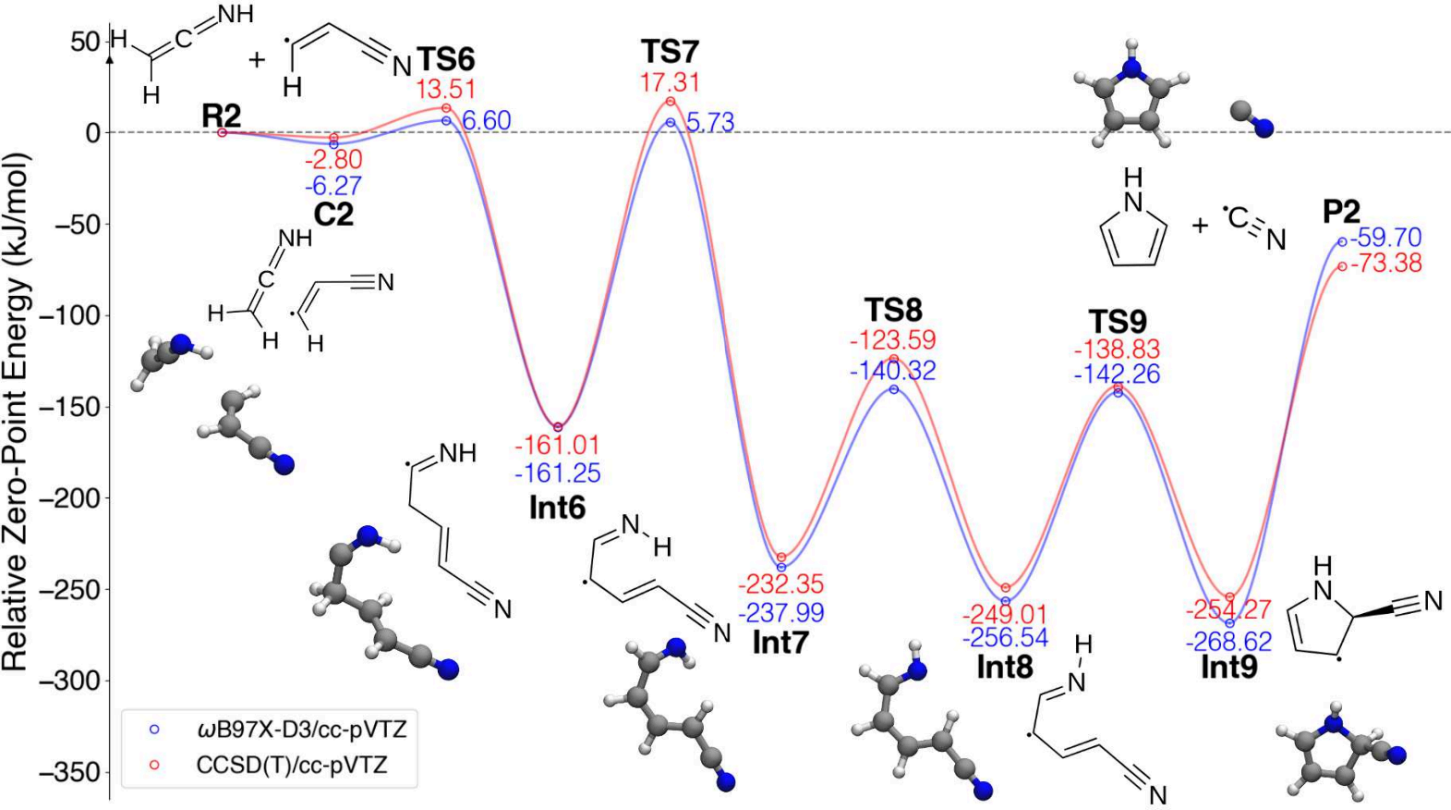
PES detailing the reaction of ethenimine (H_2_CCNH) with β-cyanovinyl (HCCHCN).

Figure 6 shows the calculated PES for the pathway **26** + **27** → pyrrole + CN. This PES was calculated on a doublet surface with singlet and doublet spin multiplicities for reactants **26** and **27**, respectively. This particular reaction coordinate was discovered by expanding the PES beyond the original nanoreactor trajectory, which proceeded through the formation of a new N–C bond between the reactants and led to several high-energy barriers. The formation of the C–C bond, as shown in Figure 6, was manually explored by altering the pathway. First, the intermediates in the nanoreactor pathway that were lower in energy than the reactants were identified. Starting with these structures, bonds were changed iteratively using Avogadro^122^ (an open-source molecular builder and visualization tool, version 1.93.0; http://avogadro.cc/) to connect the reactants and products. These new intermediates were minimized at uB3LYP/6-31G*, and a Cartesian interpolation of the coordinates was used to generate initial path estimates. These paths were then refined with NEB and TS optimizations as discussed in section 2.

Starting from reactants **R2**, the unimolecular intermediate **Int6** is formed by making a C–C bond between the two terminal carbons of the reactants. An initial prereactive complex **C2** followed by a slight barrier transition state **TS6** lies along the entrance channel. Subsequently, a hydrogen migration leads to intermediate **Int7** and forces the structure toward planarity owing to an allylic structure. The barrier to this H migration at transition state **TS7** is computed to lie 5.73 and 17.31 kJ/mol above the reactants in the DFT and CCSD(T) calculations, respectively. This is followed by a trans to cis isomerization of the terminal NH to yield intermediate **Int8**, followed by ring closure by C–N bond formation (**Int9**) and elimination of CN^•^ to yield pyrrole.

This pathway has a number of similarities to the pyridine formation mechanism from β-cyanovinyl and vinyl cyanide which was calculated to be effectively barrierless at the uB3LYP/cc-pVTZ//CCSD(T)/cc-pVTZ level of theory.^26^ In both cases the reaction is initiated with the formation of a C–C bond, the intermediates are all acyclic and unbranched until ring formation occurs, and the steps with the highest activation barriers involve a hydrogen migration.

Ethenimine has been detected in hot molecular cores (HMCs) within the giant molecular cloud Sagittarius B2 (Sgr B2), where it is believed to form from the tautomerization of methyl cyanide (CH_3_CN) during shocks.^123^ The barrier to tautomerization from ketenimine back to methyl cyanide is calculated to be 262 kJ/mol at the CCSD(T)/aug-cc-pVTZ level,^124^ preventing the reverse reaction from occurring spontaneously. An additional formation mechanism of ketenimine is H + cyanomethyl radical (CH_2_CN), which has been calculated to be barrierless and exothermic.^124^ CH_2_CN has also been detected in Sgr B2, as well as the Taurus Molecular Cloud 1.^116^ Vinyl cyanide, the parent species of β-cyanovinyl, has been detected in multiple environments in the ISM,^93,125^ including recently within HMC G10.47 + 0.03.^126^ While barrier heights of transition states **TS6** and **TS7** likely inhibit the reaction **26** + **27** in dense molecular clouds where temperatures typically are 10–20 K,^127^ it may be operative in HMCs as temperatures may reach 300 K.^128^

In the Solar System, vinyl cyanide was detected on Titan in 2017,^18^ and the presence of the cyanovinyl radicals is likely because photodissociation processes are rapid in the upper atmosphere. Ethenimine has not been detected directly in Titan’s atmosphere but seems likely to be present based on crossed molecular beam experiments of N(^2^D) + C_2_H_2_ which saw ethenimine form with a branching ratio between 4 and 5%.^124^ At the warmer temperatures of Titan’s atmosphere compared with dense clouds, the calculated barrier height for the reaction **26** + **27** is likely small enough that the reaction is feasible. Additional exploration of the PES and kinetic studies are needed to determine the viability of this pathway in specific environments and its branching ratio relative to other pathways on the PES.

#### 3.2.3 1-Pyrrolyl + Cyanomethylene Diradical

**Figure 7:**
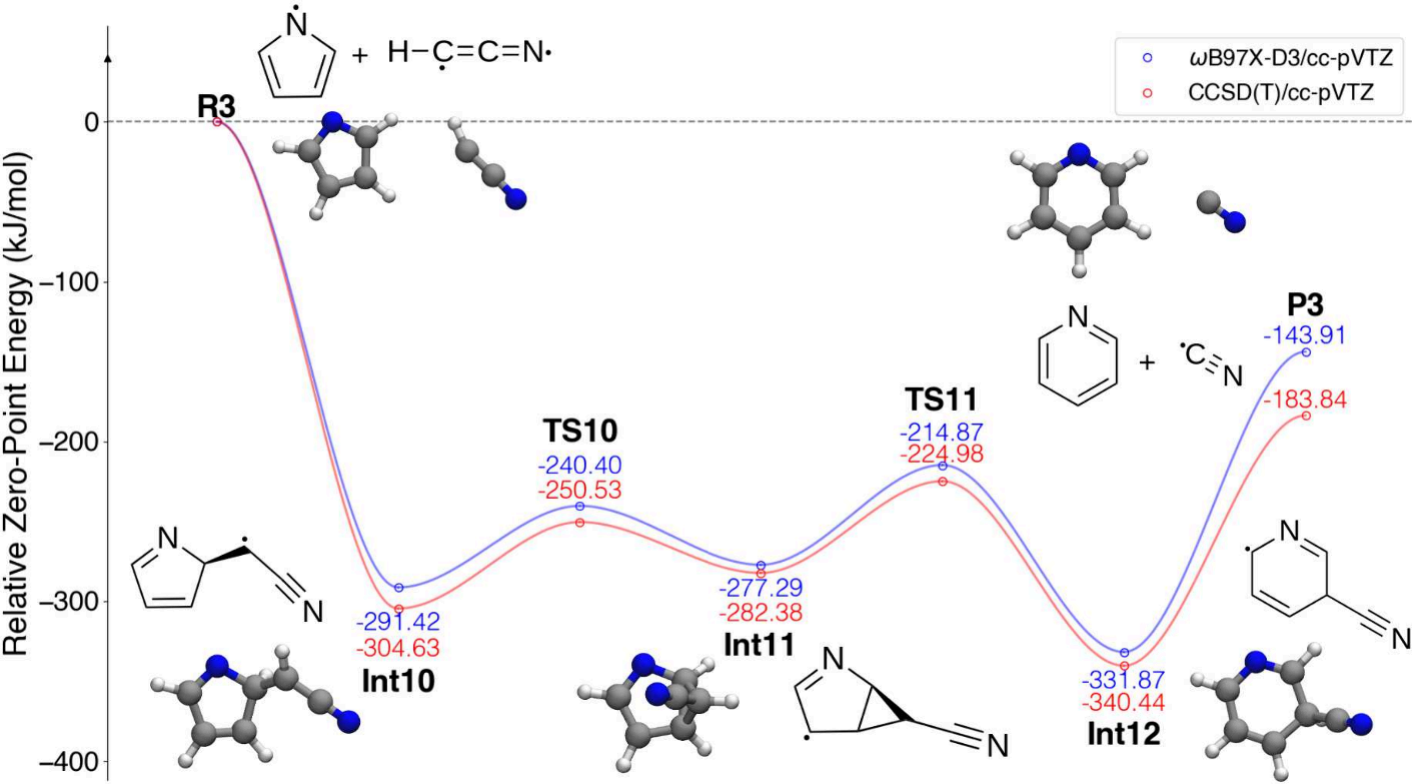
PES detailing the reaction of 1-pyrrolyl (c-C_4_H_4_N) with the cyanomethylene diradical (HCCN^••^).

The PES for the reaction **13** + **14** → pyridine + CN is shown in Figure 7. The calculation was performed on the doublet surface but unlike the previous two reactions, in this case both reactants are open-shell. 1-Pyrrolyl is well-known to be a doublet in its electronic ground state with π-radical character.^129^ HCCN is a carbene which has been experimentally determined to have a triplet electronic ground state and quasi-linear structure.^130,131^ Its HCC bond angle has been calculated to be 145.9° at the CCSD(T)/aug-cc-pVQZ level of theory and the barrier to linearity to be only 0.67–0.86 kcal/mol.^132^ Here, we also observe a quasi-linear structure with an HCC bond angle of 153.8° at the ωB97X-D3/cc-pVTZ level. In the PES, radicals **13** and **14** were calculated with doublet and triplet spin multiplicity, respectively.

From the reactants **R3**, no transition state or prereaction complex was along the reaction coordinate leading to intermediate **Int10**, in contrast to the previouly discussed reactions. Instead, HCCN bonds directly to the α C in pyrrolyl via the terminal carbon in HCCN. This same carbon atom then forms a second bond to the adjacent pyrrole β C to reach the bicyclic structure **Int11**. The bond between the α and β C atoms then breaks and the ring widens and flattens to produce the 3-cyanopyridyl radical **Int12**, followed by elimination of CN to form pyridine. The transition states **TS10** and **TS11** are submerged relative to both reactants and products.

This reaction bears similarity to the proposed mechanism of CH insertion into pyrrole.^27^ In that case, CH first bonds concertedly to both the α and β C atoms in pyrrole, followed by breaking the C_α_–C_β_ bond and the loss of the nitrogen-bonded H. Because the reaction **13** + **14** starts from pyrrolyl, no H elimination from the N is required. Another similar reaction was detected in the pyrolysis of 3-methoxypyridine,^133^ where CO elimination from the pyridoxy radical (C_5_H_4_NO) leads to pyrrolyl. Unlike the insertion reactions with CH and HCCN, however, calculations suggest that cleavage of the O–CH_3_ bond passes through an overall barrier along the reaction coordinate.

Astronomical detections of HCCN include a definitive detection in circumstellar envelope IRC+10216 and a tentative detection in dense cloud Sgr B2.^134^ HCCN has also been suggested to be important in the formation of aerosols in Titan’s atmosphere, known as tholins, that make up the moon’s distinctive orange haze.^135^ Chemical models^120^ predict HCCN to be the third most abundant open-shell species. However, ring insertion/elimination reactions are not currently included in chemical kinetics models of Titan’s atmosphere.^119,120,136^ The 1-pyrrolyl radical has not been measured by rotationally resolved spectroscopy, precluding astronomical searches to date with radiotelescopes.

## 4 Conclusion

Here, we have demonstrated the use of an *ab initio* nanoreactor as a tool for the discovery of novel reaction pathways of astrochemical relevance, with a focus on the N-containing heterocycles pyrrole and pyridine. The key aspect of these simulations is that they begin with the heterocycle of interest coupled with an appropriate leaving group, in this case CN, and exploit the nanoreactor’s ability to stochastically explore the space of high-barrier/endothermic pathways to arrive at non-intuitive products. These same pathways, viewed in reverse, serve as potential formation pathways for the heterocycles.

In total, 249 simulations involving pyridine + CN and 156 simulations with pyrrole + CN were performed and analyzed. Nineteen simulations of pyridine and seventeen simulations of pyrrole resulted in two products. The optimal simulations for the discovery of bimolecular pathways start with only one N-heterocycle and one radical byproduct (in this case, CN) in a spherical volume containing helium atoms. Searching for pathways in this reverse manner requires that a ring breakage occur, but fragmentation into three or more components is avoided. The results suggest that this is best achieved by tuning the parameters that most directly affect the velocities of the sphere contents as they are compressed, notably *r*_1_ and *k*_2_.

Three pathways have been identified, and the PESs were calculated here at the ωB97X-D3/cc-pVTZ level of theory, with single-point energy calculations also carried out at the CCSD(T)/cc-pVTZ level of theory: the reaction **13** + **14** → pyridine + CN and the reaction **26** + **27** → pyrrole + CN which were observed directly in the nanoreactor simulations, and azete + cyanomethyl → pyrrole + CN which was inspired by radicals **14** + **31**. The ring expansion reactions **13** + **14** and azete + cyanomethyl are excellent examples of non-intuitive pathways which are barrierless or have a small barrier. These are feasible even in low-temperature regions of the ISM if the precursors are initially present, and are likely operative in the atmosphere of Titan where tentative detections of N-heterocycles have been reported. Finally, the reaction **26** + **27** leads to pyrrole from acyclic precursors through a reaction coordinate possessing a small barrier. This reaction is especially interesting as it represents a potential pathway to N-heterocycle formation directly from acyclic precursors that are likely present in hot cores as well as in Titan’s atmosphere, where the temperatures are warmer than cold interstellar clouds.

The *ab initio* nanoreactor approach employed here is readily applicable to other N-heterocycles of interest, such as pyrimidine, purine, imidazole, indole, and others, as well as functionalized derivatives. Additionally, variation of the co-product (CN in the present study) to, e.g., OH, CH, C_2_H, or others, is worthwhile. Future studies along these lines are envisioned, as well as more detailed computational investigations of the remaining pathways identified in this proof-of-concept study and integration with tools designed for automatic PES exploration.

## Supporting Information Available

Nanoreactor simulation parameters and trajectory results (PDF)
Optimized geometries of nanoreactor products, harmonic frequencies of stationary points, and internal reaction coordinate scans for transition states (ZIP)

## References

[ref1] Wöhler F. Ueber Künstliche Bildung Des Harnstoffs. Ann. Phys. 1828, 88 (2), 253–256. 10.1002/andp.18280880206.

[ref2] Eschenmoser A.; Volkan Kisakürek M. Chemistry and the Origin of Life. Helv. Chim. Acta 1996, 79, 1249–1259. 10.1002/hlca.19960790503.

[ref3] Hirao I.; Kimoto M.; Yamashige R. Natural versus Artificial Creation of Base Pairs in DNA: Origin of Nucleobases from the Perspectives of Unnatural Base Pair Studies. Acc. Chem. Res. 2012, 45, 2055–2065. 10.1021/ar200257x. 22263525

[ref4] Rios A. C.; Tor Y. On the Origin of the Canonical Nucleobases: An Assessment of Selection Pressures across Chemical and Early Biological Evolution. Isr. J. Chem. 2013, 53, 469–483. 10.1002/ijch.201300009. 25284884 PMC4181368

[ref5] Peeters Z.; Botta O.; Charnley S. B.; Ruiterkamp R.; Ehrenfreund P. The Astrobiology of Nucleobases. Astrophys. J. 2003, 593, L129 10.1086/378346.

[ref6] Martins Z. The Nitrogen Heterocycle Content of Meteorites and Their Significance for the Origin of Life. Life 2018, 8, 28 10.3390/life8030028. 29997327 PMC6160977

[ref7] Hudgins D. M.; Bauschlicher C. W. Jr.; Allamandola L. J. Variations in the Peak Position of the 6.2 μm Interstellar Emission Feature: A Tracer of N in the Interstellar Polycyclic Aromatic Hydrocarbon Population. Astrophys. J. 2005, 632 (1), 316–332. 10.1086/432495.

[ref8] Endres C. P.; Schlemmer S.; Schilke P.; Stutzki J.; Müller H. S. The Cologne Database for Molecular Spectroscopy, CDMS, in the Virtual Atomic and Molecular Data Centre, VAMDC. J. Mol. Spectrosc. 2016, 327, 95–104. 10.1016/j.jms.2016.03.005.

[ref9] Kwok S. Complex Organics in Space from Solar System to Distant Galaxies. Astron. Astrophys. Rev. 2016, 24, 8 10.1007/s00159-016-0093-y.

[ref10] McGuire B. A.; Burkhardt A. M.; Kalenskii S.; Shingledecker C. N.; Remijan A. J.; Herbst E.; McCarthy M. C. Detection of the Aromatic Molecule Benzonitrile (c-C_6_H_5_CN) in the Interstellar Medium. Science 2018, 359, 202–205. 10.1126/science.aao4890. 29326270

[ref11] Barnum T. J.; Siebert M. A.; Lee K. L. K.; Loomis R. A.; Changala P. B.; Charnley S. B.; Sita M. L.; Xue C.; Remijan A. J.; Burkhardt A. M.; McGuire B. A.; Cooke I. R. A Search for Heterocycles in GOTHAM Observations of TMC-1. J. Phys. Chem. A 2022, 126, 2716–2728. 10.1021/acs.jpca.2c01435. 35442689

[ref12] Mccarthy M. C.; Mcguire B. A. Aromatics and Cyclic Molecules in Molecular Clouds: A New Dimension of Interstellar Organic Chemistry. J. Phys. Chem. A 2021, 125, 3231–3243. 10.1021/acs.jpca.1c00129. 33749264

[ref13] Cooke I. R.; Gupta D.; Messinger J. P.; Sims I. R. Benzonitrile as a Proxy for Benzene in the Cold ISM: Low-temperature Rate Coefficients for CN + C_6_H_6_. Astrophys. J. 2020, 891, L41 10.3847/2041-8213/ab7a9c.

[ref14] Cernicharo J.; Agúndez M.; Cabezas C.; Tercero B.; Marcelino N.; Pardo J. R.; de Vicente P. Pure hydrocarbon cycles in TMC-1: Discovery of ethynyl cyclopropenylidene, cyclopentadiene, and indene. Astron. Astrophys. 2021, 649, L15 10.1051/0004-6361/202141156. 34257463 PMC7611194

[ref15] McGuire B. A.; Loomis R. A.; Burkhardt A. M.; Lee K. L. K.; Shingledecker C. N.; Charnley S. B.; Cooke I. R.; Cordiner M. A.; Herbst E.; Kalenskii S.; Siebert M. A.; Willis E. R.; Xue C.; Remijan A. J.; McCarthy M. C. Detection of two interstellar polycyclic aromatic hydrocarbons via spectral matched filtering. Science 2021, 371, 1265–1269. 10.1126/science.abb7535. 33737489

[ref16] Vuitton V.; Yelle R.; McEwan M. Ion Chemistry and N-containing Molecules in Titan’s Upper Atmosphere. Icarus 2007, 191, 722–742. 10.1016/j.icarus.2007.06.023.

[ref17] Nixon C. A.; Thelen A. E.; Cordiner M. A.; Kisiel Z.; Charnley S. B.; Molter E. M.; Serigano J.; Irwin P. G. J.; Teanby N. A.; Kuan Y.-J. Detection of Cyclopropenylidene on Titan with ALMA. Astron. J. 2020, 160, 205 10.3847/1538-3881/abb679.

[ref18] Palmer M. Y.; Cordiner M. A.; Nixon C. A.; Charnley S. B.; Teanby N. A.; Kisiel Z.; Irwin P. G. J.; Mumma M. J. ALMA Detection and Astrobiological Potential of Vinyl Cyanide on Titan. Sci. Adv. 2017, 3, e1700022 10.1126/sciadv.1700022. 28782019 PMC5533535

[ref19] Hörst S.; Yelle R.; Buch A.; Carrasco N.; Cernogora G.; Dutuit O.; Quirico E.; Sciamma-O’Brien E.; Smith M.; Somogyi Á.; Szopa C.; Thissen R.; Vuitton V. Formation of Amino Acids and Nucleotide Bases in a Titan Atmosphere Simulation Experiment. Astrobiology 2012, 12, 809–817. 10.1089/ast.2011.0623. 22917035 PMC3444770

[ref20] Trainer M. G.; Pavlov A. A.; DeWitt H. L.; Jimenez J. L.; McKay C. P.; Toon O. B.; Tolbert M. A. Organic Haze on Titan and the Early Earth. Proc. Natl. Acad. Sci. U. S. A. 2006, 103, 18035–18042. 10.1073/pnas.0608561103. 17101962 PMC1838702

[ref21] Coustenis A. Titans Atmosphere and Surface - Parallels and Differences with the Primitive Earth. Earth Moon Planets 1994, 67, 95–100. 10.1007/BF00613295.

[ref22] Wakelam V.; Loison J.-C.; Herbst E.; Pavone B.; Bergeat A.; Beroff K.; Chabot M.; Faure A.; Galli D.; Geppert W. D.; Gerlich D.; Gratier P.; Harada N.; Hickson K. M.; Honvault P.; Klippenstein S. J.; Le Picard S. D.; Nyman G.; Ruaud M.; Schlemmer S.; Sims I. R.; Talbi D.; Tennyson J.; Wester R. The 2014 KIDA Network for Interstellar Chemistry. Astrophys. J. Suppl. Ser. 2015, 217, 20 10.1088/0067-0049/217/2/20.

[ref23] Millar T. J.; Walsh C.; Van de Sande M.; Markwick A. J. The UMIST Database for Astrochemistry 2022. Astron. Astrophys. 2024, 682, A109 10.1051/0004-6361/202346908.

[ref24] Lee K. L. K. Interstellar Aromatic Chemistry: A Combined Laboratory, Observational, and Theoretical Perspective. IAU S350 Laboratory Astrophysics: From Observations to Interpretation; Cambridge University Press: Cambridge, U.K., 2019.

[ref25] McCarthy M. C.; Lee K. L. K.; Loomis R. A.; Burkhardt A. M.; Shingledecker C. N.; Charnley S. B.; Cordiner M. A.; Herbst E.; Kalenskii S.; Willis E. R.; Xue C.; Remijan A. J.; McGuire B. A. Interstellar Detection of the Highly Polar Five-Membered Ring Cyanocyclopentadiene. Nat. Astron. 2021, 5, 176–180. 10.1038/s41550-020-01213-y.

[ref26] Parker D. S. N.; Kaiser R. I.; Kostko O.; Troy T. P.; Ahmed M.; Sun B.-J.; Chen S.-H.; Chang A. H. H. On the Formation of Pyridine in the Interstellar Medium. Phys. Chem. Chem. Phys. 2015, 17, 32000–32008. 10.1039/C5CP02960K. 26569517

[ref27] Soorkia S.; Taatjes C. A.; Osborn D. L.; Selby T. M.; Trevitt A. J.; Wilson K. R.; Leone S. R. Direct Detection of Pyridine Formation by the Reaction of CH (CD) with Pyrrole: A Ring Expansion Reaction. Phys. Chem. Chem. Phys. 2010, 12, 8750–8758. 10.1039/c002135k. 20463997

[ref28] Morales S. B.; Bennett C. J.; Le Picard S. D.; Canosa A.; Sims I. R.; Sun B. J.; Chen P. H.; Chang A. H. H.; Kislov V. V.; Mebel A. M.; Gu X.; Zhang F.; Maksyutenko P.; Kaiser R. I. A Crossed Molecular Beam, Low-Temperature Kinetics, and Theoretical Investigation of the Reaction of the Cyano Radical (CN) with 1,3-Butadiene (C_4_H_6_). A Route to Complex Nitrogen-Bearing Molecules in Low-Temperature Extraterrestrial Environments. Astrophys. J. 2011, 742, 26 10.1088/0004-637X/742/1/26.

[ref29] Parker D. S.; Kaiser R. I.; Kostko O.; Troy T. P.; Ahmed M.; Mebel A. M.; Tielens A. G. Gas Phase Synthesis of (ISO)Quinoline and Its Role in the Formation of Nucleobases in the Interstellar Medium. Astrophys. J. 2015, 803, 53 10.1088/0004-637X/803/2/53.

[ref30] Zhao L.; Prendergast M.; Kaiser R. I.; Xu B.; Lu W.; Ahmed M.; Hasan Howlader A.; Wnuk S. F.; Korotchenko A. S.; Evseev M. M.; Bashkirov E. K.; Azyazov V. N.; Mebel A. M. A Molecular Beams and Computational Study on the Barrierless Gas Phase Formation of (Iso)quinoline in Low Temperature Extraterrestrial Environments. Phys. Chem. Chem. Phys. 2021, 23, 18495–18505. 10.1039/D1CP02169A. 34612388

[ref31] Recio P.; Marchione D.; Caracciolo A.; Murray V. J.; Mancini L.; Rosi M.; Casavecchia P.; Balucani N. A Crossed Molecular Beam Investigation of the N (^2^*D*) + Pyridine Reaction and Implications for Prebiotic Chemistry. Chem. Phys. Lett. 2021, 779, 138852 10.1016/j.cplett.2021.138852.

[ref32] Chin C.-H.; Zhu T.; Zhang J. Z. H. Cyclopentadienyl Radical Formation from the Reaction of Excited Nitrogen Atoms with Benzene: A Theoretical Study. Phys. Chem. Chem. Phys. 2021, 23, 12408–12420. 10.1039/D1CP00133G. 34027937

[ref33] Hamid A. M.; Bera P. P.; Lee T. J.; Aziz S. G.; Alyoubi A. O.; El-Shall M. S. Evidence for the Formation of Pyrimidine Cations from the Sequential Reactions of Hydrogen Cyanide with the Acetylene Radical Cation. J. Phys. Chem. Lett. 2014, 5, 3392–3398. 10.1021/jz501648q. 26278451

[ref34] Soliman A.-R.; Hamid A. M.; Attah I.; Momoh P.; El-Shall M. S. Formation of Nitrogen-Containing Polycyclic Cations by Gas-Phase and Intracluster Reactions of Acetylene with the Pyridinium and Pyrimidinium Ions. J. Am. Chem. Soc. 2013, 135, 155–166. 10.1021/ja3068116. 23205891

[ref35] Wang Z.-C.; Cole C. A.; Snow T. P.; Bierbaum V. M. Experimental and Computational Studies of the Formation Mechanism of Protonated Interstellar Diazines. Astrophys. J. 2015, 798, 102 10.1088/0004-637X/798/2/102.

[ref36] Zimmerman P. M. Navigating Molecular Space for Reaction Mechanisms: An Efficient, Automated Procedure. Mol. Simul. 2015, 41, 43–54. 10.1080/08927022.2014.894999.

[ref37] Bergeler M.; Simm G. N.; Proppe J.; Reiher M. Heuristics-Guided Exploration of Reaction Mechanisms. J. Chem. Theory Comput. 2015, 11, 5712–5722. 10.1021/acs.jctc.5b00866. 26642988

[ref38] Suleimanov Y. V.; Green W. H. Automated Discovery of Elementary Chemical Reaction Steps Using Freezing String and Berny Optimization Methods. J. Chem. Theory Comput. 2015, 11, 4248–4259. 10.1021/acs.jctc.5b00407. 26575920

[ref39] Martínez-Núñez E. An Automated Method to Find Transition States Using Chemical Dynamics Simulations. J. Comput. Chem. 2015, 36, 222–234. 10.1002/jcc.23790. 25413470

[ref40] Martínez-Núñez E. An Automated Transition State Search Using Classical Trajectories Initialized at Multiple Minima. Phys. Chem. Chem. Phys. 2015, 17, 14912–14921. 10.1039/C5CP02175H. 25982874

[ref41] Gao C. W.; Allen J. W.; Green W. H.; West R. H. Reaction Mechanism Generator: Automatic Construction of Chemical Kinetic Mechanisms. Comput. Phys. Commun. 2016, 203, 212–225. 10.1016/j.cpc.2016.02.013.

[ref42] Pendleton I. M.; Pérez-Temprano M. H.; Sanford M. S.; Zimmerman P. M. Experimental and Computational Assessment of Reactivity and Mechanism in C(Sp3)–N Bond-Forming Reductive Elimination from Palladium(IV). J. Am. Chem. Soc. 2016, 138, 6049–6060. 10.1021/jacs.6b02714. 27087364

[ref43] Maeda S.; Harabuchi Y.; Takagi M.; Taketsugu T.; Morokuma K. Artificial Force Induced Reaction (AFIR) Method for Exploring Quantum Chemical Potential Energy Surfaces. Chem. Rec. 2016, 16, 2232–2248. 10.1002/tcr.201600043. 27258568

[ref44] Dewyer A. L.; Zimmerman P. M. Finding Reaction Mechanisms, Intuitive or Otherwise. Org. Biomol. Chem. 2017, 15, 501–504. 10.1039/C6OB02183B. 27942662

[ref45] Kopec S.; Martínez-Núñez E.; Soto J.; Peláez D. vdW-TSSCDS—An Automated and Global Procedure for the Computation of Stationary Points on Intermolecular Potential Energy Surfaces. Int. J. Quantum Chem. 2019, 119, e26008 10.1002/qua.26008.

[ref46] Unsleber J. P.; Reiher M. The Exploration of Chemical Reaction Networks. Annu. Rev. Phys. Chem. 2020, 71, 121–142. 10.1146/annurev-physchem-071119-040123. 32105566

[ref47] Martínez-Núñez E.; Barnes G. L.; Glowacki D. R.; Kopec S.; Peláez D.; Rodríguez A.; Rodríguez-Fernández R.; Shannon R. J.; Stewart J. J. P.; Tahoces P. G.; Vazquez S. A. AutoMeKin2021: An Open-Source Program for Automated Reaction Discovery. J. Comput. Chem. 2021, 42, 2036–2048. 10.1002/jcc.26734. 34387374

[ref48] Zhao Q.; Savoie B. M. Algorithmic Explorations of Unimolecular and Bimolecular Reaction Spaces. Angew. Chem., Int. Ed. 2022, 61, e202210693 10.1002/anie.202210693. PMC9827825 36074520

[ref49] Ismail I.; Chantreau Majerus R.; Habershon S. Graph-Driven Reaction Discovery: Progress, Challenges, and Future Opportunities. J. Phys. Chem. A 2022, 126, 7051–7069. 10.1021/acs.jpca.2c06408. 36190262 PMC9574932

[ref50] Zhang G.; Li J.; Liang X.; Liu Z. Automated Reaction Mechanisms and Kinetics with the Nudged Elastic Band Method-Based AMK_Mountain and Its Description of the Preliminary Alkaline Hydrolysis of Nitrocellulose Monomer. J. Comput. Chem. 2022, 43, 1513–1523. 10.1002/jcc.26891. 35567577

[ref51] Zhao Q.; Vaddadi S. M.; Woulfe M.; Ogunfowora L. A.; Garimella S. S.; Isayev O.; Savoie B. M. Comprehensive Exploration of Graphically Defined Reaction Spaces. Sci. Data 2023, 10, 145 10.1038/s41597-023-02043-z. 36935430 PMC10025260

[ref52] Pietrucci F.; Andreoni W. Graph Theory Meets Ab Initio Molecular Dynamics: Atomic Structures and Transformations at the Nanoscale. Phys. Rev. Lett. 2011, 107, 085504 10.1103/PhysRevLett.107.085504. 21929175

[ref53] Wang L.-P.; Titov A.; McGibbon R.; Liu F.; Pande V. S.; Martínez T. J. Discovering Chemistry with an Ab Initio Nanoreactor. Nat. Chem. 2014, 6, 1044–1048. 10.1038/nchem.2099. 25411881 PMC4239668

[ref54] Wang L. P.; McGibbon R. T.; Pande V. S.; Martinez T. J. Automated Discovery and Refinement of Reactive Molecular Dynamics Pathways. J. Chem. Theory Comput. 2016, 12, 638–649. 10.1021/acs.jctc.5b00830. 26683346

[ref55] Xie L.; Zhao Q.; Jensen K. F.; Kulik H. J. Direct Observation of Early-Stage Quantum Dot Growth Mechanisms with High-Temperature Ab Initio Molecular Dynamics. J. Phys. Chem. C 2016, 120, 2472–2483. 10.1021/acs.jpcc.5b12091.

[ref56] Jara-Toro R. A.; Pino G. A.; Glowacki D. R.; Shannon R. J.; Martínez-Núñez E. Enhancing Automated Reaction Discovery with Boxed Molecular Dynamics in Energy Space. ChemSystemsChem 2020, 2, e1900024 10.1002/syst.201900024.

[ref57] Shannon R. J.; Martínez-Núñez E.; Shalashilin D. V.; Glowacki D. R. ChemDyME: Kinetically Steered, Automated Mechanism Generation through Combined Molecular Dynamics and Master Equation Calculations. J. Chem. Theory Comput. 2021, 17, 4901–4912. 10.1021/acs.jctc.1c00335. 34283599

[ref58] Cui Q.; Peng J.; Xu C.; Lan Z. Automatic Approach to Explore the Multireaction Mechanism for Medium-Sized Bimolecular Reactions via Collision Dynamics Simulations and Transition State Searches. J. Chem. Theory Comput. 2022, 18, 910–924. 10.1021/acs.jctc.1c00795. 35061380

[ref59] Hirai H.; Jinnouchi R. Discovering Surface Reaction Pathways Using Accelerated Molecular Dynamics and Network Analysis Tools. RSC Adv. 2022, 12, 23274–23283. 10.1039/D2RA04343B. 36090391 PMC9382359

[ref60] Rasmussen M. H.; Jensen J. H. Fast and Automated Identification of Reactions with Low Barriers Using Meta-MD Simulations. PeerJ. Phys. Chem. 2022, 4, e22 10.7717/peerj-pchem.22.

[ref61] Stan A.; Esch B. V. D.; Ochsenfeld C. Fully Automated Generation of Prebiotically Relevant Reaction Networks from Optimized Nanoreactor Simulations. J. Chem. Theory Comput. 2022, 18, 6700–6712. 10.1021/acs.jctc.2c00754. 36270030

[ref62] Sumiya Y.; Harabuchi Y.; Nagata Y.; Maeda S. Quantum Chemical Calculations to Trace Back Reaction Paths for the Prediction of Reactants. JACS Au 2022, 2, 1181–1188. 10.1021/jacsau.2c00157. 35647604 PMC9131471

[ref63] Robertson C.; Hyland R.; Lacey A. J. D.; Havens S.; Habershon S. Identifying Barrierless Mechanisms for Benzene Formation in the Interstellar Medium Using Permutationally Invariant Reaction Discovery. J. Chem. Theory Comput. 2021, 17, 2307–2322. 10.1021/acs.jctc.1c00046. 33730851

[ref64] Zádor J.; Martí C.; Van De Vijver R.; Johansen S. L.; Yang Y.; Michelsen H. A.; Najm H. N. Automated Reaction Kinetics of Gas-Phase Organic Species over Multiwell Potential Energy Surfaces. J. Phys. Chem. A 2023, 127, 565–588. 10.1021/acs.jpca.2c06558. 36607817

[ref65] Habershon S. Automated Prediction of Catalytic Mechanism and Rate Law Using Graph-Based Reaction Path Sampling. J. Chem. Theory Comput. 2016, 12, 1786–1798. 10.1021/acs.jctc.6b00005. 26938837

[ref66] Varela A.; Vázquez S. A.; Martínez-Núñez E. An Automated Method to Find Reaction Mechanisms and Solve the Kinetics in Organometallic Catalysis. Chem. Sci. 2017, 8, 3843–3851. 10.1039/C7SC00549K. 28966776 PMC5577717

[ref67] Gao M.; Lyalin A.; Maeda S.; Taketsugu T. Application of Automated Reaction Path Search Methods to a Systematic Search of Single-Bond Activation Pathways Catalyzed by Small Metal Clusters: A Case Study on H–H Activation by Gold. J. Chem. Theory Comput. 2014, 10, 1623–1630. 10.1021/ct500068b. 26580374

[ref68] Jafari M.; Zimmerman P. M. Uncovering Reaction Sequences on Surfaces through Graphical Methods. Phys. Chem. Chem. Phys. 2018, 20, 7721–7729. 10.1039/C8CP00044A. 29498390

[ref69] Pieri E.; Lahana D.; Chang A. M.; Aldaz C. R.; Thompson K. C.; Martínez T. J. The Non-Adiabatic Nanoreactor: Towards the Automated Discovery of Photochemistry. Chem. Sci. 2021, 12, 7294–7307. 10.1039/D1SC00775K. 34163820 PMC8171323

[ref70] Goldman N.; Reed E. J.; Fried L. E.; William Kuo I.-F.; Maiti A. Synthesis of Glycine-Containing Complexes in Impacts of Comets on Early Earth. Nat. Chem. 2010, 2, 949–954. 10.1038/nchem.827. 20966951

[ref71] Saitta A. M.; Saija F. Miller Experiments in Atomistic Computer Simulations. Proc. Natl. Acad. Sci. U. S. A. 2014, 111, 13768–13773. 10.1073/pnas.1402894111. 25201948 PMC4183268

[ref72] Zubarev D. Y.; Rappoport D.; Aspuru-Guzik A. Uncertainty of Prebiotic Scenarios: The Case of the Non-Enzymatic Reverse Tricarboxylic Acid Cycle. Sci. Rep. 2015, 5, 8009 10.1038/srep08009. 25620471 PMC4306138

[ref73] Zhao Q.; Garimella S. S.; Savoie B. M. Thermally Accessible Prebiotic Pathways for Forming Ribonucleic Acid and Protein Precursors from Aqueous Hydrogen Cyanide. J. Am. Chem. Soc. 2023, 145, 6135–6143. 10.1021/jacs.2c11857. 36883252

[ref74] Garay-Ruiz D.; Álvarez-Moreno M.; Bo C.; Martínez-Núñez E. New Tools for Taming Complex Reaction Networks: The Unimolecular Decomposition of Indole Revisited. ACS Phys. Chem. Au 2022, 2, 225–236. 10.1021/acsphyschemau.1c00051. 36855573 PMC9718323

[ref75] Martínez L.; Andrade R.; Birgin E. G.; Martínez J. M. PACKMOL: A Package for Building Initial Configurations for Molecular Dynamics Simulations. J. Comput. Chem. 2009, 30, 2157–2164. 10.1002/jcc.21224. 19229944

[ref76] Lee C.; Yang W.; Parr R. G. Development of the Colle-Salvetti Correlation-Energy Formula into a Functional of the Electron Density. Phys. Rev. B 1988, 37, 785–789. 10.1103/PhysRevB.37.785. 9944570

[ref77] Becke A. D. Density-functional Thermochemistry. III. The Role of Exact Exchange. J. Chem. Phys. 1993, 98, 5648–5652. 10.1063/1.464913.

[ref78] Ufimtsev I. S.; Martinez T. J. Quantum Chemistry on Graphical Processing Units. 3. Analytical Energy Gradients, Geometry Optimization, and First Principles Molecular Dynamics. J. Chem. Theory Comput. 2009, 5, 2619–2628. 10.1021/ct9003004. 26631777

[ref79] Humphrey W.; Dalke A.; Schulten K. VMD – Visual Molecular Dynamics. J. Mol. Graph. 1996, 14, 33–38. 10.1016/0263-7855(96)00018-5. 8744570

[ref80] Snow T. P.; McCall B. J. Diffuse Atomic and Molecular Clouds. Annu. Rev. Astron. Astrophys. 2006, 44, 367–414. 10.1146/annurev.astro.43.072103.150624.

[ref81] Lin Y.-S.; Li G.-D.; Mao S.-P.; Chai J.-D. Long-Range Corrected Hybrid Density Functionals with Improved Dispersion Corrections. J. Chem. Theory Comput. 2013, 9, 263–272. 10.1021/ct300715s. 26589028

[ref82] Shao Y.; et al. Advances in Molecular Quantum Chemistry Contained in the Q-Chem 4 Program Package. Mol. Phys. 2015, 113, 184–215. 10.1080/00268976.2014.952696.

[ref83] Kelvin Lee K. L.; Loomis R. A.; Burkhardt A. M.; Cooke I. R.; Xue C.; Siebert M. A.; Shingledecker C. N.; Remijan A.; Charnley S. B.; McCarthy M. C.; McGuire B. A. Discovery of Interstellar Trans -Cyanovinylacetylene (HCCCHCHCN) and Vinylcyanoacetylene (H_2_CCHC_3_N) in GOTHAM Observations of TMC-1. Astrophys. J. 2021, 908, L11 10.3847/2041-8213/abdbb9.

[ref84] Zdanovskaia M. A.; Dorman P. M.; Orr V. L.; Owen A. N.; Kougias S. M.; Esselman B. J.; Woods R. C.; McMahon R. J. Rotational Spectra of Three Cyanobutadiene Isomers (C_5_H_5_N) of Relevance to Astrochemistry and Other Harsh Reaction Environments. J. Am. Chem. Soc. 2021, 143, 9551–9564. 10.1021/jacs.1c03777. 34155881

[ref85] McGuire B. A.; Burkhardt A. M.; Loomis R. A.; Shingledecker C. N.; Lee K. L. K.; Charnley S. B.; Cordiner M. A.; Herbst E.; Kalenskii S.; Momjian E.; Willis E. R.; Xue C.; Remijan A. J.; McCarthy M. C. Early Science from GOTHAM: Project Overview, Methods, and the Detection of Interstellar Propargyl Cyanide (HCCCH_2_CN) in TMC-1. Astrophys. J. Lett. 2020, 900, L10 10.3847/2041-8213/aba632.

[ref86] Vrtilek J. M.; Gottlieb C. A.; Thaddeus P. Laboratory and Astronomical Spectroscopy of C_3_H_2_, the First Interstellar Organic Ring. Astrophys. J. 1987, 314, 716–725. 10.1086/165099.

[ref87] Hollis J. M.; Remijan A. J.; Jewell P. R.; Lovas F. J. Cyclopropenone (c-H_2_C_3_O): A New Interstellar Ring Molecule. Astrophys. J. 2006, 642, 933 10.1086/501121.

[ref88] Cooke I. R.; Xue C.; Changala P. B.; Shay H. T.; Byrne A. N.; Tang Q. Y.; Fried Z. T. P.; Kelvin Lee K. L.; Loomis R. A.; Lamberts T.; Remijan A.; Burkhardt A. M.; Herbst E.; McCarthy M. C.; McGuire B. A. Detection of Interstellar E-1-cyano-1,3-Butadiene in GOTHAM Observations of TMC-1. Astrophys. J. 2023, 948, 133 10.3847/1538-4357/acc584.

[ref89] Ewen H. I.; Purcell E. M. Observation of a Line in the Galactic Radio Spectrum: Radiation from Galactic Hydrogen at 1,420 Mc./Sec. Nature 1951, 168, 356–356. 10.1038/168356a0.

[ref90] RIDGWAY S. T.; HALL D. N. B.; KLEINMANN S. G.; WEINBERGER D. A.; WOJSLAW R. S. Circumstellar Acetylene in the Infrared Spectrum of IRC + 10° 216. Nature 1976, 264, 345–346. 10.1038/264345a0.

[ref91] Betz A. L. Ethylene in IRC + 10216. Astrophys. J. 1981, 244, L103 10.1086/183490.

[ref92] Lacy J. H.; Evans N. J. II; Achtermann J. M.; Bruce D. E.; Arens J. F.; Carr J. S. Discovery of Interstellar Acetylene. Astrophys. J. 1989, 342, L43 10.1086/185480.

[ref93] Gardner F. F.; Winnewisser G. The Detection of Interstellar Vinyl Cyanide (Acrylonitrile). Astrophys. J. 1975, 195, L127 10.1086/181726.

[ref94] Agúndez M.; Marcelino N.; Cernicharo J. Discovery of Interstellar Isocyanogen (CNCN): Further Evidence That Dicyanopolyynes Are Abundant in Space. Astrophys. J. 2018, 861, L22 10.3847/2041-8213/aad089. PMC6120679 30186588

[ref95] Zaleski D. P.; Seifert N. A.; Steber A. L.; Muckle M. T.; Loomis R. A.; Corby J. F.; Martinez O. Jr.; Crabtree K. N.; Jewell P. R.; Hollis J. M.; Lovas F. J.; Vasquez D.; Nyiramahirwe J.; Sciortino N.; Johnson K.; McCarthy M. C.; Remijan A. J.; Pate B. H. Detection of E-Cyanomethanimine toward Saggitarius B2(N) in the Green Bank Telescope PRIMOS Survey. Astrophys. J. Lett. 2013, 765, L10 10.1088/2041-8205/765/1/L10.

[ref96] Rivilla V. M.; Martín-Pintado J.; Jiménez-Serra I.; Zeng S.; Martín S.; Armijos-Abendaño J.; Requena-Torres M. A.; Aladro R.; Riquelme D. Abundant Z-cyanomethanimine in the Interstellar Medium: Paving the Way to the Synthesis of Adenine. Mon. Not. R. Astron. Soc. Lett. 2019, 483, L114–L119. 10.1093/mnrasl/sly228.

[ref97] Belloche A.; Menten K. M.; Comito C.; Müller H. S. P.; Schilke P.; Ott J.; Thorwirth S.; Hieret C. Detection of Amino Acetonitrile in Sgr B2(N). Astron. Astrophys. 2008, 492, 769–773. 10.1051/0004-6361:20079203e.

[ref98] Agúndez M.; Cernicharo J.; de Vicente P.; Marcelino N.; Roueff E.; Fuente A.; Gerin M.; Guélin M.; Albo C.; Barcia A.; Barbas L.; Bolaño R.; Colomer F.; Diez M. C.; Gallego J. D.; Gómez-González J.; López-Fernández I.; López-Fernández J. A.; López-Pérez J. A.; Malo I.; Serna J. M.; Tercero F. Probing Non-Polar Interstellar Molecules through Their Protonated Form: Detection of Protonated Cyanogen (NCCNH^+^). Astron. Astrophys. 2015, 579, L10 10.1051/0004-6361/201526650. 26543239 PMC4630856

[ref99] Turner B. E.; Liszt H. S.; Kaifu N.; Kisliakov A. G. Microwave Detection of Interstellar Cyanamide. Astrophys. J. 1975, 201, L149 10.1086/181963.

[ref100] McGuire B. A.; Loomis R. A.; Charness C. M.; Corby J. F.; Blake G. A.; Hollis J. M.; Lovas F. J.; Jewell P. R.; Remijan A. J. Interstellar Carbodiimide (HNCNH): A New Astronomical Detection from the Gbt PRIMOS Survey via Maser Emission Features. Astrophys. J. Lett. 2012, 758, L33 10.1088/2041-8205/758/2/L33.

[ref101] Jiang N.; Melosso M.; Bizzocchi L.; Alessandrini S.; Guillemin J.-C.; Dore L.; Puzzarini C. Spectroscopic and Computational Characterization of 2-Aza-1,3-Butadiene, a Molecule of Astrochemical Significance. J. Phys. Chem. A 2022, 126, 1881–1888. 10.1021/acs.jpca.2c00831. 35275628 PMC8958585

[ref102] Guelin M.; Thaddeus P. Tentative Detection of the C_3_N Radical. Astrophys. J. Lett. 1977, 212, L81 10.1086/182380.

[ref103] Friberg P.; Hjalmarson A.; Guelin M.; Irvine W. M. Interstellar C3N - Detection in Taurus Dark Clouds. Astrophys. J. Lett. 1980, 241, L99–L103. 10.1086/183369.

[ref104] Cernicharo J.; Agúndez M.; Cabezas C.; Marcelino N.; Tercero B.; Pardo J. R.; Gallego J. D.; Tercero F.; López-Pérez J. A.; de Vicente P. Discovery of CH_2_CHCCH and Detection of HCCN, HC_4_N, CH_3_CH_2_CN, and, Tentatively, CH_3_CH_2_CCH in TMC-1. Astron. Astrophys. 2021, 647, L2 10.1051/0004-6361/202140434. 33833468 PMC7610549

[ref105] Cernicharo J.; Cabezas C.; Agúndez M.; Tercero B.; Marcelino N.; Pardo J. R.; Tercero F.; Gallego J. D.; López-Pérez J. A.; DeVicente P. Discovery of Allenyl Acetylene, H_2_CCCHCCH, in TMC-1. Astron. Astrophys. 2021, 647, L3 10.1051/0004-6361/202140482. 33850332 PMC7610584

[ref106] Fuentetaja R.; Cabezas C.; Agúndez M.; Tercero B.; Marcelino N.; Pardo J. R.; de Vicente P.; Cernicharo J. Discovery of CH_2_CCHC_4_H and a Rigorous Detection of CH_2_CCHC_3_N in TMC-1 with the QUIJOTE Line Survey. Astron. Astrophys. 2022, 663, L3 10.1051/0004-6361/202243857.

[ref107] Heitkämper J.; Suchaneck S.; García de la Concepción J.; Kästner J.; Molpeceres G. The Reactivity of Pyridine in Cold Interstellar Environments: The Reaction of Pyridine with the CN Radical. Front. Astron. Space Sci. 2022, 9, 1020635 10.3389/fspas.2022.1020635.

[ref108] Esselman B. J.; Kougias S. M.; Zdanovskaia M. A.; Woods R. C.; McMahon R. J. Synthesis, Purification, and Rotational Spectroscopy of (Cyanomethylene)Cyclopropane—An Isomer of Pyridine. J. Phys. Chem. A 2021, 125, 5601–5614. 10.1021/acs.jpca.1c03246. 34153184

[ref109] Loomis R. A.; Zaleski D. P.; Steber A. L.; Neill J. L.; Muckle M. T.; Harris B. J.; Hollis J. M.; Jewell P. R.; Lattanzi V.; Lovas F. J.; Martinez O.; McCarthy M. C.; Remijan A. J.; Pate B. H.; Corby J. F. The Detection of Interstellar Ethanimine (CH_3_CHNH) from Observations Taken during the GBT PRIMOS Survey. Astrophys. J. Lett. 2013, 765, L9 10.1088/2041-8205/765/1/L9.

[ref110] Godfrey P. D.; Brown R. D.; Robinson B. J.; Sinclair M. W. Discovery of Interstellar Methanimine (Formaldimine). Astrophys. Lett. 1973, 13, 119–121.

[ref111] Zeng S.; Jiménez-Serra I.; Rivilla V. M.; Martín-Pintado J.; Rodríguez-Almeida L. F.; Tercero B.; de Vicente P.; Rico-Villas F.; Colzi L.; Martín S.; Requena-Torres M. A. Probing the Chemical Complexity of Amines in the ISM: Detection of Vinylamine (C_2_H_3_NH_2_) and Tentative Detection of Ethylamine (C_2_H_5_NH_2_). Astrophys. J. Lett. 2021, 920, L27 10.3847/2041-8213/ac2c7e. PMC7611195 34257894

[ref112] Motiyenko R. A.; Margulès L.; Guillemin J.-C. Millimeter- and Submillimeter-Wave Spectrum of Methyleneaminoacetonitrile. Astron. Astrophys. 2013, 559, A44 10.1051/0004-6361/201322371.

[ref113] Luková K.; Kolesniková L.; Koucký J.; Vávra K.; Kania P.; Guillemin J.-C.; Urban Š. Decoding Millimetre-Wave Spectra of 2-Iminopropanenitrile, a Candidate for Astronomical Observations. Astron. Astrophys. 2022, 665, A9 10.1051/0004-6361/202243696.

[ref114] Bonačić-Koutecký V.; Schöffel K.; Michl J. Electronic States of Cyclobutadiene Heteroanalogues. Critical Biradicaloids. J. Am. Chem. Soc. 1989, 111, 6140–6146. 10.1021/ja00198a024.

[ref115] Császár A. G.; Demaison J.; Rudolph H. D. Equilibrium Structures of Three-, Four-, Five-, Six-, and Seven-Membered Unsaturated N-containing Heterocycles. J. Phys. Chem. A 2015, 119, 1731–1746. 10.1021/jp5084168. 25340501

[ref116] Irvine W. M.; Friberg P.; Hjalmarson A.; Ishikawa S.; Kaifu N.; Kawaguchi K.; Madden S. C.; Matthews H. E.; Ohishi M.; Saito S.; Suzuki H.; Thaddeus P.; Turner B. E.; Yamamoto S.; Ziurys L. M. Identification of the Interstellar Cyanomethyl Radical (CH_2_CN) in the Molecular Clouds TMC-1 and Sagittarius B2. Astrophys. J. 1988, 334, L107 10.1086/185323. 11538463

[ref117] Waite J. H.; Niemann H.; Yelle R. V.; Kasprzak W. T.; Cravens T. E.; Luhmann J. G.; McNutt R. L.; Ip W.-H.; Gell D.; De La Haye V.; Müller-Wordag I.; Magee B.; Borggren N.; Ledvina S.; Fletcher G.; Walter E.; Miller R.; Scherer S.; Thorpe R.; Xu J.; Block B.; Arnett K. Ion Neutral Mass Spectrometer Results from the First Flyby of Titan. Science 2005, 308, 982–986. 10.1126/science.1110652. 15890873

[ref118] Waite J. H.; Young D. T.; Cravens T. E.; Coates A. J.; Crary F. J.; Magee B.; Westlake J. The Process of Tholin Formation in Titan’s Upper Atmosphere. Science 2007, 316, 870–875. 10.1126/science.1139727. 17495166

[ref119] Vuitton V.; Yelle R.; Klippenstein S.; Hörst S.; Lavvas P. Simulating the Density of Organic Species in the Atmosphere of Titan with a Coupled Ion-Neutral Photochemical Model. Icarus 2019, 324, 120–197. 10.1016/j.icarus.2018.06.013.

[ref120] Lavvas P. P.; Coustenis A.; Vardavas I. M. Coupling Photochemistry with Haze Formation in Titan’s Atmosphere, Part II: Results and Validation with Cassini/Huygens Data. Planet. Space Sci. 2008, 56, 67–99. 10.1016/j.pss.2007.05.027.

[ref121] Maier G.; Schäfer U. Kleine Ringe, 27. Versuche Zur Darstellung von Azacyclobutadienen. Lieb. Ann. Chem. 1980, 1980, 798–813. 10.1002/jlac.198019800520.

[ref122] Hanwell M. D.; Curtis D. E.; Lonie D. C.; Vandermeersch T.; Zurek E.; Hutchison G. R. Avogadro: An Advanced Semantic Chemical Editor, Visualization, and Analysis Platform. J. Cheminf. 2012, 4, 17 10.1186/1758-2946-4-17. PMC3542060 22889332

[ref123] Lovas F. J.; Hollis J. M.; Remijan A. J.; Jewell P. R. Detection of Ketenimine (CH_2_CNH) in Sagittarius B2(N) Hot Cores. Astrophys. J. 2006, 645, L137–L140. 10.1086/506324.

[ref124] Balucani N.; Skouteris D.; Leonori F.; Petrucci R.; Hamberg M.; Geppert W. D.; Casavecchia P.; Rosi M. Combined Crossed Beam and Theoretical Studies of the N ^2^*D* + C_2_H_4_ Reaction and Implications for Atmospheric Models of Titan. J. Phys. Chem. A 2012, 116, 10467–10479. 10.1021/jp3072316. 23016665

[ref125] Matthews H. E.; Sears T. J. The Detection of Vinyl Cyanide in TMC-1. Astrophys. J. 1983, 272, 149 10.1086/161271.

[ref126] Mondal S. K.; Iqbal W.; Gorai P.; Bhat B.; Wakelam V.; Das A. Investigating the Hot Molecular Core, G10.47 + 0.03: A Pit of Nitrogen-Bearing Complex Organic Molecules. Astron. Astrophys. 2023, 669, A71 10.1051/0004-6361/202243802.

[ref127] Bergin E. A.; Tafalla M. Cold Dark Clouds: The Initial Conditions for Star Formation. Annu. Rev. Astron. Astrophys. 2007, 45, 339–396. 10.1146/annurev.astro.45.071206.100404.

[ref128] Garrod R. T.; Widicus Weaver S. L. Simulations of Hot-Core Chemistry. Chem. Rev. 2013, 113, 8939–8960. 10.1021/cr400147g. 24024866

[ref129] Johansen S. L.; Xu Z.; Westerfield J. H.; Wannenmacher A. C.; Crabtree K. N. Coupled Cluster Characterization of 1-, 2-, and 3-Pyrrolyl: Parameters for Vibrational and Rotational Spectroscopy. J. Phys. Chem. A 2021, 125, 1257–1268. 10.1021/acs.jpca.0c09833. 33502858

[ref130] Brown F. X.; Saito S.; Yamamoto S. Microwave Spectroscopy of Isotopically Substituted HCCN and Its Molecular Structure. J. Mol. Spectrosc. 1990, 143, 203–208. 10.1016/0022-2852(91)90084-N.

[ref131] Bernheim R. A.; Kempf R. J.; Gramas J. V.; Skell P. S. Electron Paramagnetic Resonance of Triplet Alternant Methylenes. Propargylene and Homologs. J. Chem. Phys. 1965, 43, 196–200. 10.1063/1.1696454.

[ref132] Abbott B. Z.; Hoobler P. R.; Schaefer H. F. Relatives of Cyanomethylene: Replacement of the Divalent Carbon by B^–^, N^+^, Al^–^, Si, P^+^, Ga^–^, Ge, and As^+^. Phys. Chem. Chem. Phys. 2019, 21, 26438–26452. 10.1039/C9CP05777C. 31774089

[ref133] Holzmeier F.; Wagner I.; Fischer I.; Bodi A.; Hemberger P. Pyrolysis of 3-Methoxypyridine. Detection and Characterization of the Pyrrolyl Radical by Threshold Photoelectron Spectroscopy. J. Phys. Chem. A 2016, 120, 4702–4710. 10.1021/acs.jpca.5b10743. 26698131

[ref134] Guelin M.; Cernicharo J. Astronomical Detection of the HCCN Radical. Toward a New Family of Carbon-Chain Molecules?. Astron. Astrophys. 1991, L21–L24.

[ref135] Lavvas P.; Sander M.; Kraft M.; Imanaka H. Surface Chemistry and Particle Shape: Processes for the Evolution of Aerosols in Titan’s atmosphere. Astrophys. J. 2011, 728, 80 10.1088/0004-637X/728/2/80.

[ref136] Krasnopolsky V. A. A Photochemical Model of Titan’s Atmosphere and Ionosphere. Icarus 2009, 201, 226–256. 10.1016/j.icarus.2008.12.038.

