# Supplementary material for: Reactant Discovery with an Ab Initio Nanoreactor: Exploration of Astrophysical N-Heterocycle Precursors and Formation Pathways
Source: ACS Earth Space Chem. 2024 Aug 9;8(9):1771–83. doi: 10.1021/acsearthspacechem.4c00120 (PMC11418024; doi:10.1021/acsearthspacechem.4c00120)
Supplement: Supplementary file 1 — sp4c00120_si_001.pdf [file sp4c00120_si_001.pdf]

Reactant discovery with an ab initio nanoreactor: Exploration of astrophysical N-heterocycle precursors and formation pathways

Sommer L. Johansen,[†,‡,¶] Heejune Park,[†,¶] Lee-Ping Wang,[*,†] and Kyle N. Crabtree[*,†]

†*Department of Chemistry, University of California, Davis, Davis, CA 95616*

‡*Present Address: Sandia National Laboratories, Livermore, CA*

¶*Equal contribution*

E-mail: leeping@ucdavis.edu; kncrabtree@ucdavis.edu

List of Tables

S1 Starting spheres for nanoreactor simulations S3

S2 Nanoreactor sphere parameters S3

S3 Bimolecular pyridine trajectories S4

S4 Bimolecular pyrrolyl trajectories S4

S5 File descriptions S5

This document is accompanied by computational input/output files in a zip archive for all of the stationary points described in the main manuscript. The contents are organized into 4 folders:

nanoreactor_products xyz files with ωB97X-D/cc-pVTZ optimized geometries of nanoreactor products. Each file is labeled by its numeric identifier in Figures 3 and 4.

reaction1 Optimized geometries (ωB97X-D/cc-pVTZ), harmonic frequencies (ωB97X-D/cc-pVTZ), and CCSD(T)/cc-pVTZ//ωB97X-D/cc-pVTZ energies of stationary points for the azete + cyanomethyl radical reaction (Figure 5). Files for each stationary point are included in a folder labeled by the structure identifier in Figure 5. Transition state structure folders also contain outputs of internal reaction coordinate (IRC) calculations connecting the structure to adjacent structures on the potential energy surface.

reaction2 Optimized geometries (ωB97X-D/cc-pVTZ), harmonic frequencies (ωB97X-D/cc-pVTZ), and CCSD(T)/cc-pVTZ//ωB97X-D/cc-pVTZ energies of stationary points for the ethenimine + β=cyanovinyl radical reaction (Figure 6). Files for each stationary point are included in a folder labeled by the structure identifier in Figure 6. Transition state structure folders also contain outputs of internal reaction coordinate (IRC) calculations connecting the structure to adjacent structures on the potential energy surface.

reaction3 Optimized geometries (ωB97X-D/cc-pVTZ), harmonic frequencies (ωB97X-D/cc-pVTZ), and CCSD(T)/cc-pVTZ//ωB97X-D/cc-pVTZ energies of stationary points for the 1-pyrrolyl + cyanomethylene diradical reaction (Figure 7). Files for each stationary point are included in a folder labeled by the structure identifier in Figure 7. Transition state structure folders also contain outputs of internal reaction coordinate (IRC) calculations connecting the structure to adjacent structures on the potential energy surface.

Table S1: Starting spheres for nanoreactor simulations. For each sphere, numbers of each species, the number of different starting configurations, and the total number of simulations are listed.

Label	Type	N-heterocycle	CN	He	Configurations	Total Simulations
Prdn1	pyridine	1	1	50	4	76
Prdn2a	pyridine	2	1	10	2	72
Prdn2b	pyridine	2	1	20	4	52
Prdn3	pyridine	3	3	20	4	25
Prdn5	pyridine	5	5	20	4	24
Pyrl1	pyrrole	1	1	50	6	60
Pyrl2	pyrrole	2	2	30	4	96

Table S2: Ranges of parameters explored for each sphere (by label in Table S1).

Sphere	T (K)	r_1 (Å)	r_2 (Å)	t_1 (0.5 fs)	t_2 (0.5 fs)	k_1 $(\frac{\text{kcal}}{\text{mol}^{-1}\text{Å}^{-2}})$	k_2 $(\frac{\text{kcal}}{\text{mol}^{-1}\text{Å}^{-2}})$
Prdn1	1500 - 2500	8.0 - 12.0	3.0 - 5.0	2000	200 - 1000	1.0	0.5 - 0.75
Prdn2a	2000	8.0 - 12.0	3.0 - 5.0	1000 - 2000	200 - 800	1.0	0.5
Prdn2b	1000 - 3500	12.0	3.0 - 5.0	2000	1000	1.0	0.5
Prdn3	1000 - 3000	12.0	4.5 - 5.0	2000	1000	1.0	0.5
Prdn5	1000 - 3000	12.0	5.0	2000	1000	1.0	0.5
Pyrl1	1500 - 3500	12.0	3.0	1500	200 - 1000	1.0	0.5 - 1.0
Pyrl2	1500 - 2500	8.0 - 12.0	3.0 - 4.0	1500	300 - 600	1.0	0.5

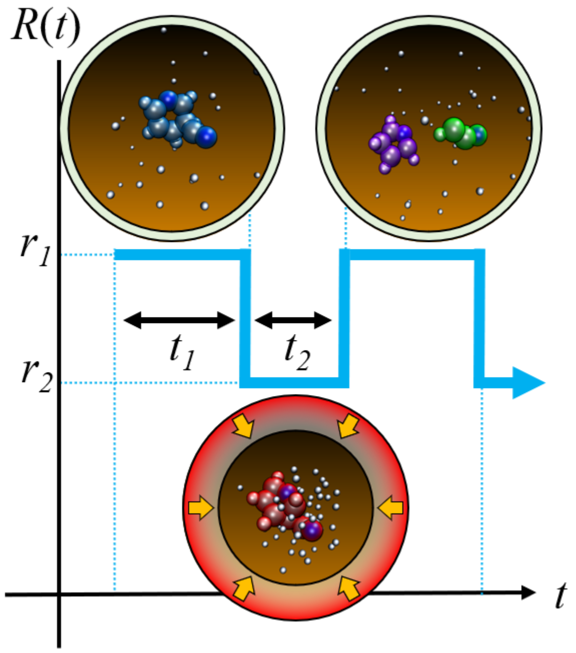

Figure S1: The visual example of the overall nanoreactor cycle.

Table S3: Parameters of Prdn1 trajectories resulting in in two products.

config	T (K)	r_1 (Å)	r_2 (Å)	k_1 $(\frac{\text{kcal}}{\text{mol}\text{Å}^2})$	k_2 $(\frac{\text{kcal}}{\text{mol}\text{Å}^2})$	t_1 (0.5 fs)	t_2 (0.5 fs)	Products
0	1500	10.0	3.0	1.0	0.5	2000	500	HCCHCHCHNC + HCN
0	2500	8.0	5.0	1.0	0.5	2000	500	HCN + para-pyridyl
0	2500	8.0	3.0	1.0	0.5	2000	500	HCN + para-pyridyl
0	2500	10.0	5.0	1.0	0.5	2000	500	H + NCCHCHCHCHCN
1	1500	12.0	3.0	1.0	0.5	2000	1000	HNC + HCCCHCHCHN
1	1500	12.0	3.0	1.0	0.6	2000	1000	HCN + $CNCHCHCCH_2$
1	2000	12.0	3.0	1.0	0.75	2000	200	H2CCNCHCCH + HCN
1	2500	8.0	3.0	1.0	0.5	2000	500	HCN + m-pyridyl
1	2500	10.0	3.0	1.0	0.5	2000	500	N_2 + HCCCHCHCHCH
1	2500	10.0	5.0	1.0	0.5	2000	500	HCCH + HCNCHCHCN
2	1500	10.0	3.0	1.0	0.5	2000	500	HCN + o-pyridyl
2	1500	12.0	3.0	1.0	0.5	2000	1000	HNC + HCCHCHCHCN
2	1500	12.0	3.0	1.0	0.6	2000	500	HCN + $HCCCHNCCH_2$
3	1500	12.0	3.0	1.0	0.75	2000	500	HCCCHCHCNH + HCN
3	1500	12.0	3.0	1.0	0.75	2000	200	pyrrolyl + HCCN
3	1500	12.0	3.0	1.0	0.75	2000	500	pyrrolyl + HCCN
3	2000	12.0	3.0	1.0	0.75	2000	200	HCN + HCCHCCHCHN
3	2500	12.0	3.0	1.0	0.5	2000	500	C_3N + $CH_2CHCHNH$
3	2500	10.0	3.0	1.0	0.5	2000	500	H + o-isocyanopyridine

Table S4: Parameters of Pyrl1 trajectories resulting in two products.

config	T (K)	r_1 (Å)	r_2 (Å)	k_1 $(\frac{\text{kcal}}{\text{mol}\text{Å}^2})$	k_2 $(\frac{\text{kcal}}{\text{mol}\text{Å}^2})$	t_1 (0.5 fs)	t_2 (0.5 fs)	Products
0	2500	12.0	3.0	1.0	0.5	1500	1000	HCN + $HCHCH_2CCN$
0	2500	12.0	3.0	1.0	0.5	1500	500	HNC + 1-pyrrolyl
0	1500	12.0	3.0	1.0	0.5	1500	1000	HCN + c-CHCHC(CHNH)
0	1500	12.0	3.0	1.0	0.5	1500	500	HCN + CCHNHCHCH
1	1500	12.0	3.0	1.0	0.5	1500	1000	HCCH + NCCHCHNH
2	1500	12.0	3.0	1.0	0.5	1500	1000	HCN + 2-pyrrolyl
3	1500	12.0	3.0	1.0	0.5	1500	500	HCCH + H2CNCHCN
3	1500	12.0	3.0	1.0	0.75	1500	200	HCCHCN + H_2CCNH
3	1500	12.0	3.0	1.0	0.75	1500	500	HCCH + NCCHCHNH
3	2500	12.0	3.0	1.0	0.5	1500	1000	H + 3-cyanopyrrole
3	3500	12.0	3.0	1.0	0.5	1500	1000	H + c-CHC(H)(HCNC)C(CN)
4	3500	12.0	3.0	1.0	0.5	1500	1000	H + 2-cyanopyrrole
5	1500	12.0	3.0	1.0	0.5	1500	500	HCCN + 1-hydroazete
5	1500	12.0	3.0	1.0	0.75	1500	200	HNC + C_4H_4N
5	1500	12.0	3.0	1.0	0.75	1500	500	2-pyrrolyl + HNC
5	2500	12.0	3.0	1.0	0.5	1500	1000	HCN + 1-pyrrolyl
5	2500	12.0	3.0	1.0	0.5	1500	500	HCN + 1-pyrrolyl

Table S5: Descriptions of files for each structure contained in folders “reaction1”, “reaction2”, and “reaction3”

Filename	Description
ccsd_sp.out	CCSD(T)/cc-pVTZ//ωB97X-D/cc-pVTZ single-point output file
freq.out	ωB97X-D/cc-pVTZ harmonic frequency calculation output file
optimize.out	ωB97X-D/cc-pVTZ geometry optimiztion output file
optimized.xyz	ωB97X-D/cc-pVTZ optimized geometry, xyz format
irc.out	ωB97X-D/cc-pVTZ internal reaction coordinate output file (TS only)
irc.xyz	ωB97X-D/cc-pVTZ optimized geometries along internal reaction coordinate (TS only)
